# Etiologic diagnosis of suspected tuberculous meningitis by multiplex PCR of cerebrospinal fluid

**DOI:** 10.1128/jcm.00446-26

**Published:** 2026-08-20

**Authors:** Haohao Wang, Mingxiang Huang, Jiabao Song, Jinding Zhang, Shangying Yang, Xiaoxing Zhang, Yongzhen He, Yiqun Liao, Ye Xu, Qingge Li

**Affiliations:** 1 Engineering Research Centre of Molecular Diagnostics of the Ministry of Education, Xiamen Key Laboratory of Multi-Target and Automated Genetic Testing, School of Life Sciences, Xiamen University 199198, Xiamen, Fujian, China; 2 Fuzhou Pulmonary Hospital 638885, Fuzhou, Fujian, China; 3 Department of Clinical Laboratory, The Second Affiliated Hospital of Fujian University of Traditional Chinese Medicine 605542, Fuzhou, Fujian, China; 4 State Key Laboratory of Vaccines for Infectious Diseases, Xiang’an Biomedicine Laboratory, Xiamen, Fujian, China; Children's Hospital Los Angeles, Los Angeles, California, USA

**Keywords:** tuberculous meningitis, multiplex PCR, MeltArray, cerebrospinal fluid, rapid diagnostics, *Mycobacterium tuberculosis*, mNGS, biomarker differentiation

## Abstract

Distinguishing tuberculous meningitis (TBM) from other causes of meningitis remains challenging in high-burden settings because clinical features overlap and existing assays often delay etiologic confirmation. We evaluated MeltArray CNS, a highly multiplexed PCR assay targeting 85 meningitis-associated pathogens, for etiologic diagnosis in patients with suspected meningitis. Analytical performance was assessed using pre-characterized targets. MeltArray was compared head-to-head with metagenomic next-generation sequencing (mNGS) in 79 cerebrospinal fluid (CSF) specimens, with discrepant results adjudicated by Sanger sequencing, and was then prospectively evaluated in 255 consecutive patients with suspected meningitis. Biomarker-based models using routine CSF and serum parameters were also explored. The assay achieved limits of detection of 5 copies/reaction for *Mycobacterium tuberculosis* (MTB) and 50 copies/reaction for other targets, with no false-positive results in analytical specificity testing. Within its targeted range, MeltArray yielded more confirmed detections than mNGS. In the prospective cohort, MeltArray showed 100.00% sensitivity for definite TBM (35/35; 95% confidence interval [CI], 90.11–100.00%) and 100.00% specificity for MTB detection among non-TBM meningitis cases (39/39; 95% CI, 89.32–100.00%). Among 49 MTB-positive TBM cases, 21 (42.86%) showed co-detections of additional pathogens. Test areas under the curve (AUCs) were 0.877 (95% CI, 0.760–0.994) for distinguishing infectious meningitis from noninfectious mimics and 0.776 (95% CI, 0.617–0.934) for distinguishing MTB-positive from MTB-negative cases. MeltArray enables rapid etiologic confirmation and may facilitate earlier TBM diagnosis in high-burden settings. Co-detections highlight microbiologic complexity with potential treatment implications, although viral findings should be interpreted cautiously. Biomarker models may aid triage and risk stratification but do not replace pathogen confirmation.

**IMPORTANCE:**

Tuberculous meningitis (TBM) is a life-threatening infection that requires a rapid and accurate diagnosis to guide effective treatment. Conventional diagnostic methods are often slow or insufficiently sensitive, leading to delays in therapy and potential exposure to unnecessary medications. In this study, we evaluated a rapid multiplex molecular assay for patients with suspected tuberculous meningitis. Rapid detection of *Mycobacterium tuberculosis* together with alternative infectious causes of meningitis was achieved within approximately 2.5 h, supporting earlier etiologic clarification during initial clinical evaluation. Detection of additional pathogens in some patients further supported the value of broad-spectrum molecular testing in the differential diagnosis of central nervous system infections in high-burden settings. Routine laboratory biomarkers may assist clinical triage but do not replace rapid pathogen confirmation.

## INTRODUCTION

Meningitis causes substantial global mortality, yet a causative pathogen remains unidentified in many patients despite extensive diagnostic evaluation (1, 2). Overlapping clinical phenotypes across infectious and noninfectious etiologies, limited sensitivity of routine microbiology, and delayed turnaround of advanced testing contribute to diagnostic uncertainty and hinder timely, targeted therapy (3). Nucleic-acid-based testing complements culture by detecting low-abundance microbial and viral nucleic acids in cerebrospinal fluid (CSF) (4, 5). Because CSF collection requires lumbar puncture, and specimen volume is often limited, sequential single-plex testing is frequently impractical. A syndromic, multiplex approach applied to total CSF nucleic acids can, therefore, provide early etiologic clarification from a single specimen across bacterial, fungal, viral, and mycobacterial meningitides.

A syndromic, multiplex approach is particularly relevant for tuberculous meningitis (TBM) (6–9). In TB-endemic, high-HIV/TB settings, TBM is often among the leading causes of adult meningitis and can comprise >30% of cases in hospital-based cohorts; it is also among the highest case-fatality rates across meningitis etiologies (10, 11). Clinical presentations of TBM are often nonspecific, and CSF profiles are insufficiently discriminatory, while microbiologic confirmation remains insensitive (7, 12). Conventional TBM diagnostics, such as CSF acid-fast bacilli (AFB) smear microscopy and mycobacterial culture, have limited sensitivity and prolonged turnaround times, further delaying etiologic confirmation (13). As a result, clinicians may rely on clinical probability, which can lead to empiric anti-tuberculous therapy in patients without microbiologic evidence and may delay appropriate treatment for competing diagnoses (14, 15). Although a highly sensitive MTB-only assay provides strong rule-in evidence when positive, negative results are common and can be difficult to interpret given low CSF organism burden, variability in sampling volume and timing, and prior antimicrobial exposure. In this context, detection of an alternative pathogen compatible with the clinical syndrome can reduce the likelihood of TBM and help direct more appropriate therapy. However, despite the availability of several syndromic tests for central nervous system infections, highly multiplexed assays that include MTB remain limited, leaving an important diagnostic gap (16).

In this study, we sought to improve etiologic diagnosis in patients with suspected tuberculous meningitis by applying the MeltArray CNS assay, a rapid CSF-based multiplex PCR assay designed for syndromic diagnosis of central nervous system infections. The assay detects *Mycobacterium tuberculosis* (MTB) alongside alternative infectious causes of meningitis within approximately 2.5 h, enabling earlier etiologic clarification and differential diagnosis during initial evaluation. To contextualize assay findings in this clinical setting, we performed an analytical evaluation and, in a subset of samples, used metagenomic next-generation sequencing (mNGS) as an orthogonal comparator to support interpretation of detection profiles. We then applied the assay in a prospective cohort of patients with suspected meningitis to assess clinical utility, including the etiologic spectrum, patterns of co-detection among MTB-positive TBM cases, and the adjunctive value of routinely available CSF biomarkers for triage and risk stratification.

## MATERIALS AND METHODS

### Study design overview

This study comprised three interconnected components: (i) analytical evaluation of the MeltArray CNS assay using reference strains and clinical isolates; (ii) head-to-head comparison with mNGS in a subset of 79 CSF samples; and (iii) prospective clinical evaluation in a consecutive cohort comprising 255 patients with suspected meningitis. The primary endpoint was MeltArray sensitivity for TBM across consensus clinical diagnostic categories. Secondary endpoints included pathogen detection rates, co-detection patterns, and the performance of biomarker-based predictive models.

### The MeltArray CNS assay procedure

The MeltArray CNS assay is based on a previously published multiplex PCR platform (17) and utilizes a multiplex panel for the detection of central nervous system pathogens in CSF. CSF (1 mL) samples underwent nucleic acid extraction using the Lab-Aid 824 system (Zeesan Biotech, Xiamen, Fujian, China) with a commercial cell-free DNA/RNA extraction kit (Zeesan Biotech) according to the manufacturer’s instructions, yielding both DNA and RNA suitable for subsequent analysis. Extracted nucleic acid (5 µL) was added to each of five multiplex PCR reactions, with reactions A–C targeting bacterial and fungal pathogens and reactions D–E targeting viral pathogens, collectively covering 85 meningitis-associated pathogens (Table S1). Amplifications were performed on a Slan-96S real-time PCR system (Hongshi, Shanghai, China) with integrated multicolor melting curve analysis (MMCA) in the Atto 425, FAM, HEX, ROX, and Cy5 fluorescence channels. The thermal protocol comprised: 50°C for 2 min, 95°C for 5 min, followed by 45 cycles of 95°C for 25 s and 63°C for 1 min (fluorescence acquisition), immediately followed by a high-resolution melting step from 45°C to 95°C at 0.16°C/s with continuous fluorescence acquisition, allowing pathogen-specific melting profiles to be analyzed. Melting curve profiles were automatically analyzed according to predefined interpretation rules.

### Analytical evaluation

Limits of detection (LODs) were established using 10-fold serial dilutions of synthetic plasmid DNA (1–10³ copies/μL; Amoy Nucleotide Biotech, Xiamen, Fujian, China) to ensure reproducibility. The LOD was defined as the lowest concentration achieving ≥95% positivity in 20 replicates. For MTB, the LOD was additionally evaluated using genomic DNA extracted from cultured MTB strains following the same 10-fold serial dilution scheme, with additional lower-concentration dilutions (down to 0.1 copies/μL) included to refine LOD estimation. Reproducibility was assessed across 24 replicates in three independent batches at a concentration of 10² copies/μL, with variation expressed as the standard deviation of each target’s melting temperature. The concentrations of DNA were measured using an ND-1000 spectrophotometer (NanoDrop Technologies, Wilmington, DE, USA).

Analytical sensitivity and specificity were evaluated using 615 archived bacterial isolates (33 species) recovered from both CSF and non-CSF clinical specimens at Zhongshan Hospital of Xiamen University between 3 January 2018 and 6 December 2018 (Table S2). In addition, 10 non-tuberculous mycobacteria (NTM) species, including *Mycobacterium avium*, *Mycobacterium intracellulare*, *Mycobacterium abscessus*, *Mycobacterium fortuitum*, *Mycobacterium kansasii*, *Mycobacterium chelonae*, *Mycobacterium gordonae*, *Mycobacterium xenopi*, *Mycobacterium scrofulaceum*, and *Mycobacterium marinum*, each represented by two clinical isolates, were included to evaluate potential cross-reactivity with NTM in MTB-specific detection. Eighteen clinical fungal isolates, including 13 within and five outside the detection scope of the MeltArray assay, were obtained from the First Affiliated Hospital of Xiamen University and identified by matrix-assisted laser desorption/ionization time-of-flight mass spectrometry (MALDI-TOF MS; Bruker Daltonics, Billerica, MA, USA). A total of 66 pre-characterized bronchoalveolar lavage fluid (BALF) specimens positive for target viruses or *Mycoplasma pneumoniae* were obtained from Xiamen Maternity and Child Health Care Hospital. Collectively, these samples were used for analytical performance evaluation of the assay. Bacterial isolates were identified using MALDI-TOF MS as the reference standard. Fungal cross-reactivity was assessed against non-target species obtained from clinical reference collections. Viral and *M. pneumoniae* targets were tested in parallel with commercially available reference assays (Zeesan Biotech), and results were confirmed where necessary by Sanger sequencing (Sangon Biotech, Shanghai, China). Quantitative accuracy was assessed for eight plasmid DNA targets across 10¹–10⁵ copies/μL, each tested in triplicate, with linear regression on quantification cycle (Cq) values to validate assay linearity and dynamic range. All samples were de-identified and used in accordance with approval from the Research Ethics Committee of Xiamen University (XDYX2021016).

### Clinical validation: mNGS comparison

CSF samples from 79 patients with suspected meningitis were tested using both the MeltArray CNS assay and in-house mNGS, with discordant results resolved by Sanger sequencing (Sangon Biotech) using previously described primers and amplification conditions. For mNGS, DNA libraries were prepared using the Rapid Plus DNA Lib Prep Kit V2 (ABclonal Technology Co., Ltd., Wuhan, Hubei, China). All libraries were quantified using a Qubit 3.0 Fluorometer (Thermo Fisher Scientific Inc., Waltham, MA, USA) and analyzed for size distribution by capillary electrophoresis on a Qsep100 Nucleic Acid Analyzer (BiOptic Inc., Taiwan, China). Indexed libraries were pooled and sequenced on a CYGNUS S100 Genetic Sequencer (Cygnus Biosciences Co., Ltd., Beijing, China) with a single-end 75 bp (SE75) strategy, ensuring robust and reproducible pathogen detection in CSF specimens. Bioinformatic analysis followed standard pipelines (fastp, BWA, Kraken2/Bracken). Detection thresholds adhered to established protocols, with microbial reads normalized as reads per million (RPM) and thresholds applied separately for bacteria, fungi, viruses, and *M. pneumoniae* as previously described (18, 19).

### Prospective clinical cohort

Between April 2023 and November 2025, 255 patients with suspected meningitis were consecutively enrolled at Fuzhou Pulmonary Hospital, China. Patients’ baseline demographics, core clinical presentation, and outcomes were extracted from electronic medical records to ensure completeness for primary analyses. Meningitis was defined by standard clinical and CSF criteria or discharge diagnosis. Standard CSF criteria included pleocytosis (CSF leukocyte count >5 × 10⁶/L) combined with at least one clinical manifestation, such as headache, fever, vomiting, neck stiffness, irritability, focal neurological deficits, convulsions, altered consciousness, or lethargy. Patients were further classified into definite TBM (microbiologically proven), probable TBM (score ≥10 points), possible TBM (score 6-9 points), or non-TBM (score <6 points) per consensus clinical scoring (20). All patients underwent testing with MeltArray and conventional microbiological methods, including culture on blood and chocolate agar for bacteria (24–48 h), Sabouraud dextrose agar for fungi (48–72 h), acid-fast bacillus (AFB) smear microscopy, and a commercial MTB-specific real-time PCR assay (*Mycobacterium tuberculosis* Complex Nucleic Acid Detection Kit; DaAn Gene, Guangzhou, Guangdong, China); parallel culture in BD BACTEC MGIT 960 and Löwenstein-Jensen medium was maintained for 6–8 weeks. Discordant results were resolved by Sanger sequencing (Sangon Biotech) using previously described methods. The sample size was calculated to ensure adequate statistical power for estimating diagnostic sensitivity of the MeltArray CNS assay for TBM.

### Biomarker modeling

Biomarkers were compared using Mann–Whitney *U* tests. Least absolute shrinkage and selection operator (LASSO) logistic regression was applied to significant univariate predictors to construct multivariable models, with the regularization parameter selected via fivefold cross-validation on the training set. The cohort was split into training (70%) and test (30%) sets using a stratified sampling approach by disease status. Model discrimination was assessed via receiver operating characteristic (ROC) curve analysis with area under the curve (AUC) and 95% confidence intervals (CIs) generated through bootstrap resampling (1,000 resamples). Optimal thresholds were determined by maximizing Youden’s index. All analyses were conducted using Python 3.12 and GraphPad Prism 9.5.

## RESULTS

### Analytical performance of the MeltArray CNS assay

**Fig 1:**
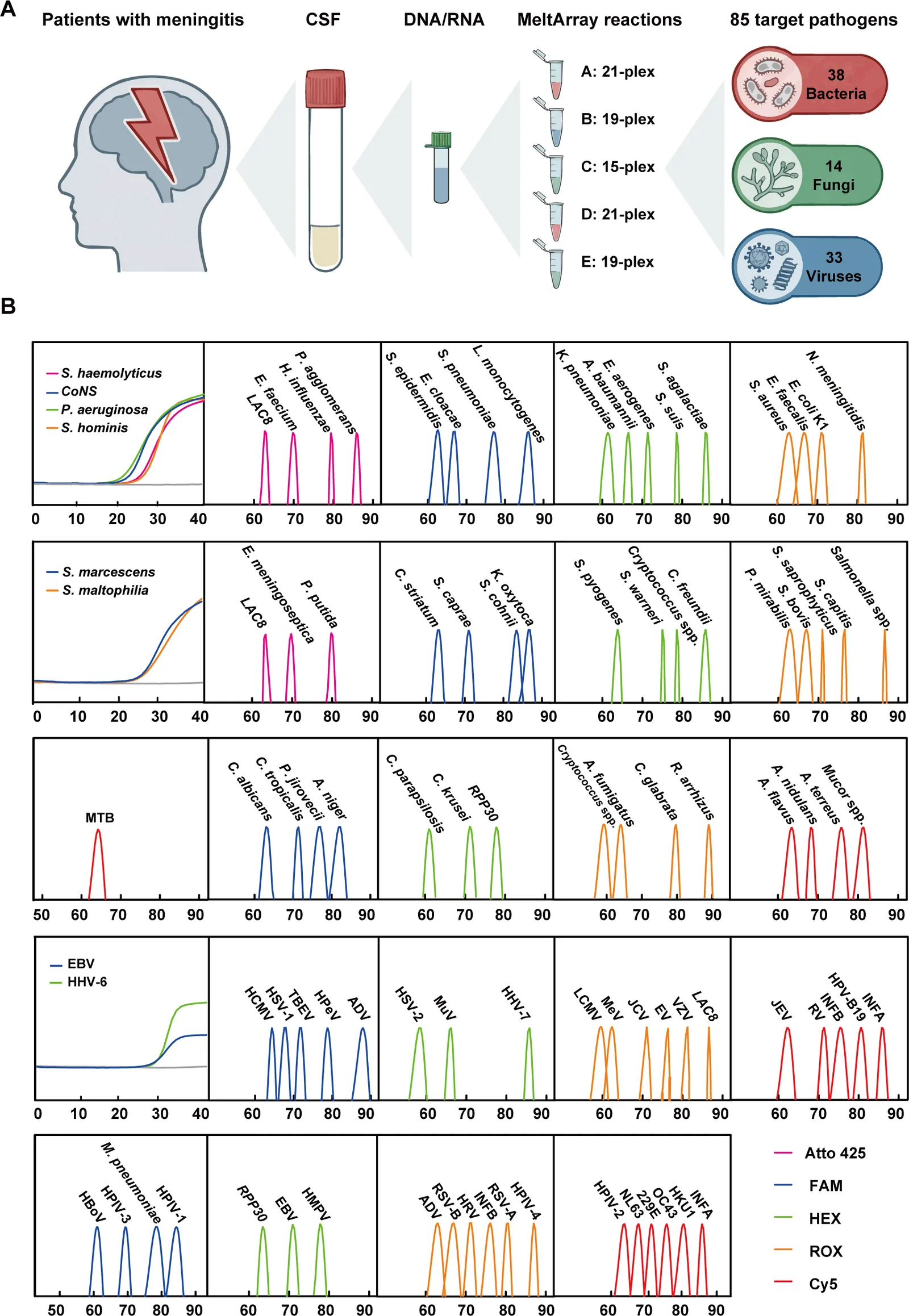
Pathogen coverage and detection strategy of the MeltArray CNS assay. (**A**) The MeltArray CNS assay was designed to simultaneously detect 85 clinically relevant CNS pathogens (comprising 38 bacteria, 14 fungi, and 33 viruses) across five multiplex PCR reactions (reactions A−E). (**B**) Schematic overview of five multiplex PCR reactions analyzed by real-time PCR with melting curve analysis. Reactions A and B collectively cover bacterial targets, with Reaction B also including *Cryptococcus* spp. for verification. Reaction C is dedicated to fungal targets. Reactions D and E collectively cover viral targets; Reaction E also includes the bacterial pathogen *Mycoplasma pneumoniae* incorporated due to primer compatibility. Pathogen-specific abbreviations above amplification and melting curves correspond to the full names and target details listed in Table S1. In plots, the *x*-axis of amplification curves represents the PCR cycle number, and the *x*-axis of melting curves represents the melting temperature (*T*_m_).

The MeltArray CNS assay was designed to simultaneously detect 85 clinically relevant CNS pathogens comprising 38 bacteria, 14 fungi, and 33 viruses across five MeltArray reactions (Fig. 1; Table S1). The entire workflow comprised nucleic acid extraction (0.5 h), followed by the MeltArray reaction step (2 h), yielding a total turnaround time of approximately 2.5 h for 1–18 samples processed on a 96-well Slan system.

The MeltArray CNS assay achieved an LOD of 5 copies/reaction for MTB and 50 copies/reaction for all other targets when using plasmid DNA as templates. Additional evaluation using genomic DNA extracted from cultured MTB strains demonstrated a lower LOD of 0.5 copies/reaction compared with plasmid DNA (five copies/reaction). This difference may reflect the multicopy nature of the target sequence within the MTB genome, leading to multiple amplifiable targets per genome equivalent. Reproducibility assessment across 24 replicates in three independent batches demonstrated melting temperature (*T*_m_) standard deviations of ≤0.2°C for all targets (Fig. S1A).

The assay demonstrated 100.00% sensitivity against 615 archived clinical isolates (33 bacterial species) and 13 reference fungal strains, with no cross-reactivity among five non-target fungal species (Fig. S1B; Table S2). No cross-reactivity was observed across the 10 NTM species. All 66 pre-characterized BALF specimens positive for viruses or *M. pneumoniae* were correctly identified, with complete concordance with commercial reference assays (Table S2). Quantitative accuracy was confirmed for eight plasmid DNA targets across 10¹–10⁵ copies/µL, with linear regression analysis yielding *R*² values ranging from 0.985 to 0.998.

### Head-to-head comparison with mNGS

**Fig 2:**
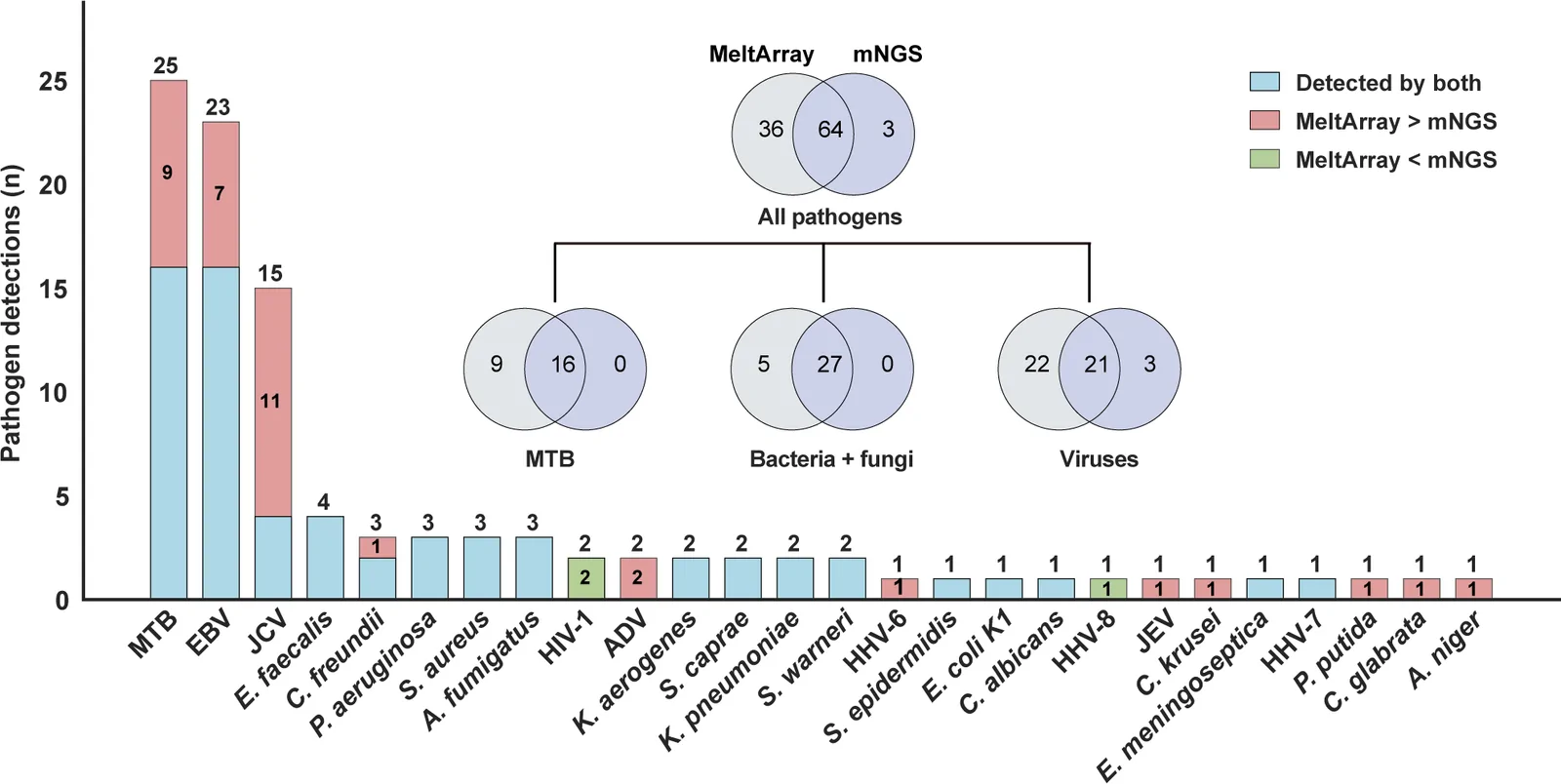
Concordance and discordance between the MeltArray CNS assay and mNGS for pathogen detection in 79 CSF samples. Discordant detections were resolved by Sanger sequencing. All pathogens detected exclusively by MeltArray (within its 85-target panel) were confirmed, demonstrating its high specificity for intended targets. Pathogens detected exclusively by mNGS were also confirmed and represent organisms outside the clinically focused MeltArray panel. Bar height represents the total number of positive detections for each pathogen by either method, stratified by method agreement: concordant detections by both assays (blue), detections exclusive to the MeltArray CNS assay (red, all confirmed by Sanger sequencing), and detections exclusive to mNGS (green, all confirmed by Sanger sequencing; targets not included in the MeltArray panel). The inset Venn diagrams quantify the overall agreement for specific pathogen categories: all pathogens, MTB, bacteria and fungi, and viruses. Because individual samples could yield multiple pathogens, the total number of detections exceeds the number of patients.

Among 79 CSF samples tested in parallel, MeltArray and mNGS showed complete concordance in 49 (62.03%) samples. MeltArray identified an additional 36 pathogens missed by mNGS, including 22 viruses, nine MTB, three fungi, and two non-MTB bacteria; all discordant results were confirmed by Sanger sequencing. Conversely, mNGS uniquely detected viruses not included in the MeltArray panel in three cases (HIV-1, *n* = 2; HHV-8, *n* = 1) (Fig. 2; Table S3). Within its targeted scope, MeltArray yielded more confirmed pathogen detections than the in-house mNGS workflow.

### Prospective clinical evaluation

**Fig 3:**
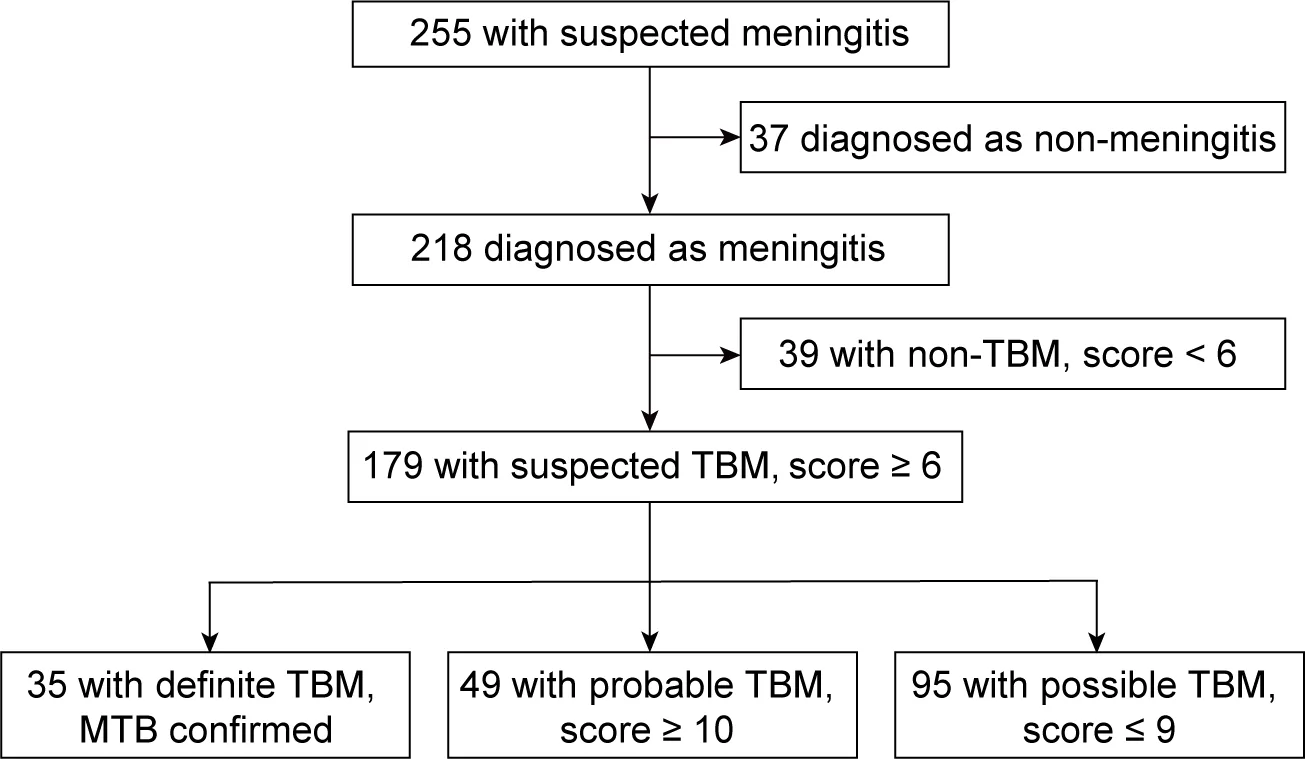
Flowchart of patient classification. A consecutive cohort of 255 patients with suspected meningitis was enrolled. Using predefined clinical diagnostic criteria, 218 patients were classified as having meningitis, while 37 did not meet these criteria and were diagnosed as non-meningitis. The 218 patients with meningitis were then evaluated against consensus clinical criteria for TBM. This identified 179 patients with suspected TBM (diagnostic score ≥ 6) and 39 patients who did not meet the TBM criteria (non-TBM, diagnostic score < 6). Finally, the 179 patients with suspected TBM were subclassified into definite (*n* = 35), probable (*n* = 49), and possible (*n* = 95) TBM categories. Definite TBM required microbiological confirmation of MTB from CSF by conventional microbiological tests (as defined in the *Methods*). Probable and possible TBM were defined by a diagnostic score of ≥10 and ≤9, respectively.

Patient characteristics are summarized in Table 1. A total of 255 consecutive patients with clinically suspected meningitis were enrolled. Of these, 37 patients (14.51%) did not meet the predefined clinical diagnostic criteria for meningitis and were classified as non-meningitis mimics; these patients served as a control group for assessing assay specificity. The remaining 218 patients constituted the meningitis cohort for diagnostic evaluation. Based on consensus clinical scoring for TBM, 39 (17.89%) were classified as non-TBM, including patients with cryptococcal, viral, or bacterial meningitis, and 179 (82.11%) were classified as suspected TBM; the latter were further categorized as possible (*n* = 95), probable (*n* = 49), and definite (*n* = 35) TBM (Fig. 3). Among the 49 probable TBM cases, 16 (32.65%) had microbiological evidence of tuberculosis from non-CSF specimens (e.g., sputum or BALF). In addition, 33 (67.35%) showed radiological findings suggestive of pulmonary tuberculosis.

**TABLE 1:** Demographics and clinical characteristics of 218 patients with suspected meningitis

Characteristic	Suspected tuberculous meningitis (*n* = 179)	Non-tuberculous meningitis (*n* = 39)	*P* value^*a*^
Age, median (range), y	53 (35–65)	42 (22–59)	0.009
Sex, no. of males/females	108/71	31/8	0.033
CSF parameter tests			
Glucose (mmol/L)	3.24 (2.73–3.87)	3.58 (3.20–4.22)	0.075
Chloride (mmol/L)	122.00 (117.40–124.30)	123.20 (121.38–125.25)	0.119
Protein (mg/L)	712.90 (457.20–1,187.20)	423.95 (306.35–538.30)	0.001
Leukocyte (10^6/L)	12.00 (3.00–47.00)	3.50 (2.00–7.25)	0.003
Lymphocyte (%)	94.10 (75.00–100.00)	100.00 (62.53–100.00)	0.341
Neutrophil (%)	5.90 (0.00–25.00)	0.00 (0.00–37.47)	0.364
Inflammatory markers			
C-reactive protein (mg/L)	8.49 (5.00–30.44)	7.77 (5.84–51.41)	0.353
Procalcitonin (ng/mL)	0.08 (0.04–0.18)	0.07 (0.04–0.24)	0.533
Clinical symptoms			
Fever	117 (65.36%)	11 (28.21%)	<0.001
Headache	66 (36.87%)	3 (7.69%)	0.001
Altered mental status	33 (18.44%)	5 (12.82%)	0.545
Neck stiffness	16 (8.94%)	3 (7.69%)	1.000
Imaging findings			
Contrast-enhanced MRI: leptomeningeal enhancement	55 (30.73%)	3 (7.69%)	0.006

^*a*^ The values are presented as medians (interquartile ranges) or *n* (%) unless otherwise stated. The *P* value compares the suspected tuberculous meningitis group (*n* = 179) and the non-tuberculous meningitis group (*n* = 39). Continuous variables were analyzed using the nonparametric Mann–Whitney *U* test, while categorical variables were assessed using Pearson’s *χ* test.

**Fig 4:**
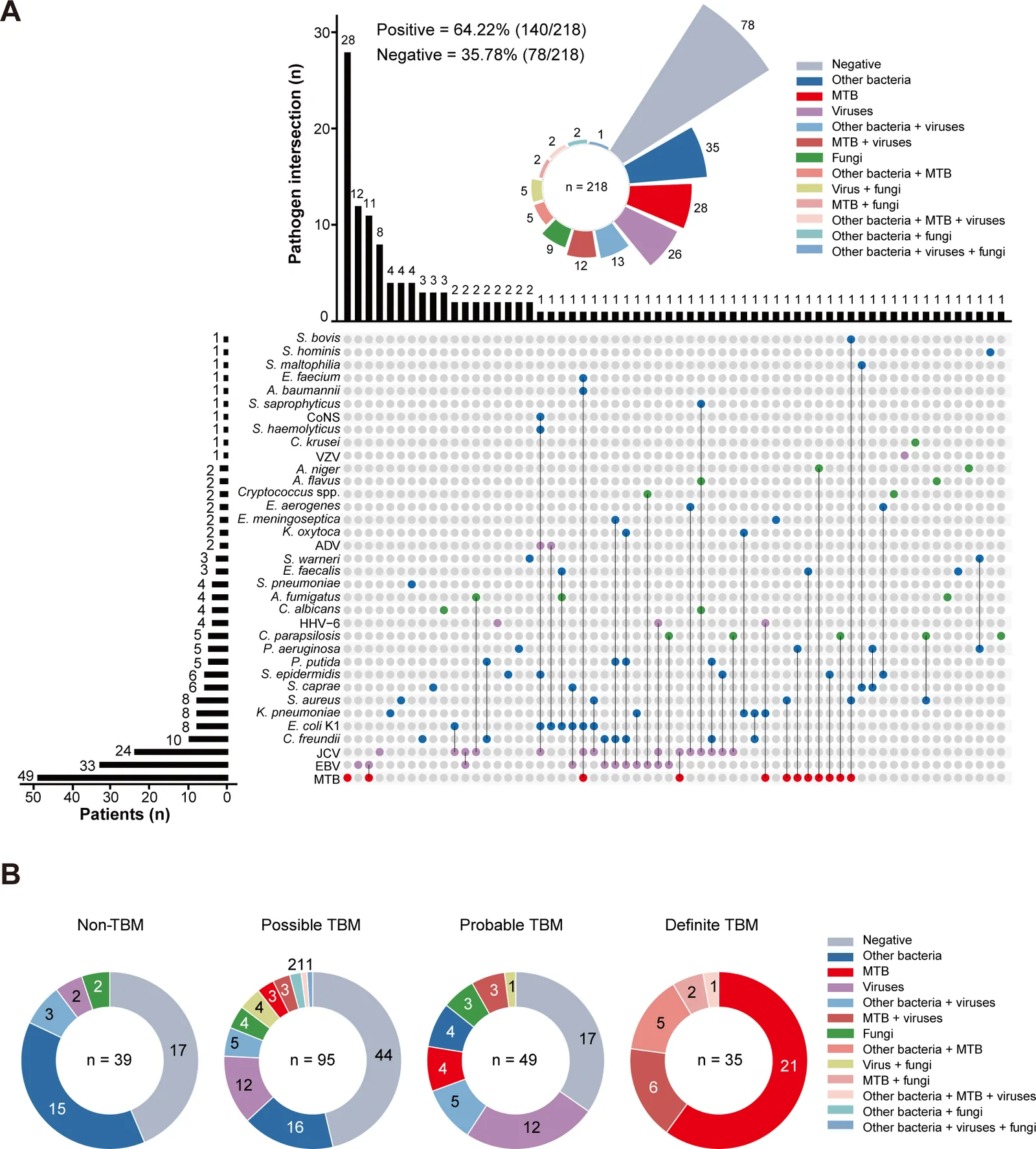
Pathogen detection and distribution across clinical subgroups of suspected meningitis. (**A**) UpSet plot illustrating the detection frequencies of 35 distinct pathogens among the 218 patients with suspected meningitis, highlighting co-detection patterns. Color coding: red, *Mycobacterium tuberculosis*; purple, viral pathogens; blue, other bacterial pathogens; and green, fungal pathogens. Overall positivity rate: 140/218 (64.22%). (**B**) CSF pathogen detection profiles stratified by diagnostic category (non-tuberculous meningitis, possible TBM, probable TBM, definite TBM).

No pathogens were detected in CSF samples from the 37 non-meningitis mimics, supporting assay specificity. At least one pathogen was detected in 140 of 218 meningitis cases, yielding an overall positivity rate of 64.22% (95% CI, 57.61–70.51%; Fig. 4A). Positivity rates did not differ significantly among non-TBM meningitis (56.41%, 22/39), possible TBM (53.68%, 51/95), and probable TBM (65.31%, 32/49) (*P* = 0.442, Fisher–Freeman–Halton test), whereas definite TBM had a positivity rate of 100.00%. After excluding MTB detections, positivity rates were 56.41% (22/39), 50.53% (48/95), 57.14% (28/49), and 40.00% (14/35) in non-TBM meningitis, possible TBM, probable TBM, and definite TBM, respectively, with no significant difference across groups (*P* = 0.374, Fisher–Freeman–Halton test).

MTB detection rates increased with diagnostic certainty, from 0% (0/39) in non-TBM to 7.37% (7/95) in possible TBM, 14.29% (7/49) in probable TBM, and 100.00% (35/35) in definite TBM (Fig. 4B), demonstrating a positive association between MTB detection rate and TBM diagnostic certainty. Using consensus clinical TBM categories as the clinical reference framework, the MeltArray CNS assay showed higher diagnostic sensitivity than conventional microbiological methods. In definite TBM cases (*n* = 35), MeltArray sensitivity was 100.00% (95% CI, 90.11–100.00%), significantly higher than culture (8.57%, *P* < 0.001) and AFB smear microscopy (11.43%, *P* < 0.001), and non-inferior to MTB-specific PCR (91.43%, *P* = 0.125) (Table 2; Table S4). Among the combined definite and probable TBM cases (*n* = 84), which represent a clinically relevant target population, MeltArray maintained the highest sensitivity at 50.00% (95% CI, 39.54–60.46%), again outperforming culture (3.57%, *P* < 0.001), AFB smear microscopy (4.76%, *P* < 0.001), and MTB-specific PCR (38.10%, *P* = 0.004). Clinical specificity for MTB detection in non-TBM meningitis was 100.00% (39/39).

**TABLE 2:** Diagnostic performance of different methods in patients with definite and probable TBM^*a*^

	Definite TBM cases (*n* = 35)			Definite and probable TBM cases (*n* = 84)		
	Sensitivity	Difference (95% CI)	*P* value	Sensitivity	Difference (95% CI)	*P* value
Mycobacterial culture	8.57% (2.96–22.38); 3/35	91.43% (77.62–97.04)	<0.001	3.57% (1.22–9.98); 3/84	46.43% (34.16–57.15)	<0.001
Acid-fast bacilli staining	11.43% (4.54–25.95); 4/35	88.57% (74.05–95.46)	<0.001	4.76% (1.87–11.61); 4/84	45.24% (32.74–56.09)	<0.001
MTB-specific PCR	91.43% (77.62–97.04); 32/35	8.57% (2.96–22.38)	0.125	38.10% (28.45–48.78); 32/84	11.90% (−3.05–26.13)	0.004
MeltArray	100.00% (90.11–100.00); 35/35	–^*b*^	–	50.00% (39.54–60.46); 42/84	–	–

^*a*^ Data are presented as percentages (95% CIs) with numerators/denominators. Analysis was restricted to patients classified as definite TBM (*n* = 35) or probable TBM (*n* = 49) according to consensus clinical diagnostic criteria; the combined group (definite and probable TBM, *n* = 84) was used for overall sensitivity assessment. Within each group, sensitivity for each diagnostic method was defined as the proportion of patients testing positive by that method. Differences in sensitivity relative to MeltArray and their 95% CIs were estimated using paired-sample comparisons; CIs for differences were computed using the Newcombe method based on Wilson score intervals. *P* values were derived from McNemar’s test with continuity correction to assess statistical significance of sensitivity differences (significance threshold, *P* < 0.05). Additional methodological notes: The commercial MTB-specific PCR assay was performed with 20 µL input of extracted nucleic acid per the manufacturer’s protocol. CI, confidence interval; MTB, *Mycobacterium tuberculosis*.

^*b*^ –, not applicable.

MTB co-detections with other pathogens were common across all TBM clinical categories. In definite TBM (*n* = 35), 21 cases (60.00%) had MTB monoinfection, and 14 cases (40.00%) had co-detections, including viral (*n* = 6, 17.14%), bacterial (*n* = 5, 14.29%), fungal (*n* = 2, 5.71%), and concurrent bacterial–viral co-detections (*n* = 1, 2.86%). Among MTB-positive cases in probable/possible TBM (*n* = 14), seven (50.00%) had MTB monoinfection, and seven (50.00%) had co-detections, comprising viral (*n* = 6, 42.86%) and concurrent bacterial-viral co-detections (*n* = 1, 7.14%). Overall, among 49 MTB-positive TBM cases, 28 (57.14%) were MTB monoinfections, and 21 (42.86%) had co-detections, including viral (*n* = 12, 24.49%), bacterial (*n* = 5, 10.20%), fungal (*n* = 2, 4.08%), and concurrent bacterial–viral co-detections (*n* = 2, 4.08%).

Among MTB co-detection cases (*n* = 21), HIV antibody testing was performed in 13 patients, all of whom were negative (0/13 [0%]). One patient was receiving methotrexate therapy. Reduced CD4^+^ T-cell counts (<550 cells/µL) were observed in 16/21 patients (76.19%). In MTB monoinfection cases (*n* = 28), HIV antibody testing was performed in 10 patients, all of whom were negative (0/10 [0%]). One patient had diabetes mellitus. Reduced CD4^+^ T-cell counts were observed in 16/28 patients (57.14%). No statistically significant difference in the proportion of reduced CD4^+^ T-cell counts was observed between groups (OR = 2.36; *P* = 0.23).

### Biomarker analysis and diagnostic model development

**Fig 5:**
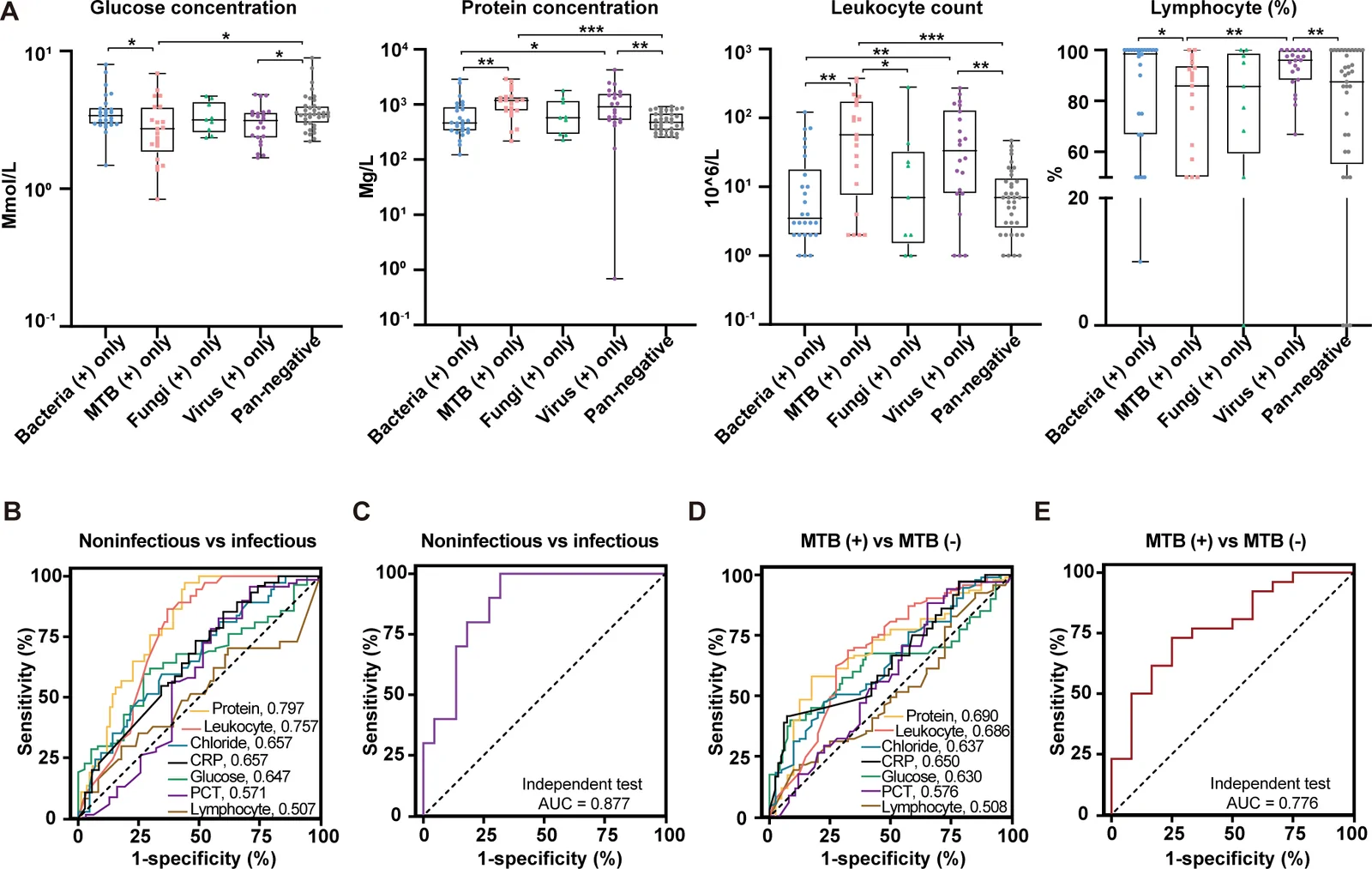
Distinct CSF and serum biomarker profiles across meningitis etiologies and development of diagnostic models. (**A**) CSF biomarker distributions stratified by etiology: bacterial meningitis, *Mycobacterium tuberculosis*-positive, fungal meningitis, viral meningitis, and pan-negative (no pathogen detected). Parameters: glucose, protein, leukocyte count, and lymphocyte percentage. Data presented as box plots (median, IQR; whiskers, 1.5× IQR). Statistical significance assessed by Mann–Whitney *U* test (*P < 0.05, **P < 0.01, ***P < 0.001). (**B**) Receiver operating characteristic (ROC) curves for discriminating noninfectious mimics from infectious meningitis using individual biomarkers. (**C**) ROC curve of the optimal model distinguishing noninfectious mimics from infectious meningitis in the independent test cohort (AUC = 0.877). The Youden index threshold (probability ≥0.258) achieved 100.00% sensitivity in the test cohort. In this cohort, probabilities <0.258 were associated with high sensitivity for excluding infectious meningitis; however, this threshold requires external validation before clinical rule-out use. (**D**) ROC curves of individual biomarkers for discriminating *Mycobacterium tuberculosis*-positive patients from *Mycobacterium tuberculosis*-negative patients. (**E**) ROC curve of the optimal model distinguishing *Mycobacterium tuberculosis*-positive patients from *Mycobacterium tuberculosis*-negative patients in the test cohort (AUC = 0.776). The high-specificity threshold (probability ≥0.779) associated with this model provides a conservative criterion for ruling in MTB infection. (+), pathogen detected by MeltArray CNS assay; (–), no pathogen detected.

To explore pathogen-specific CSF biomarker profiles, we first focused on patients with mono-etiologic meningitis. Five mono-etiologic cohorts classified by the MeltArray CNS assay showed statistically significant intergroup differences in CSF glucose, protein, leukocyte count, lymphocyte percentage, chloride, and serum C-reactive protein (CRP) (all *P* < 0.05), whereas serum procalcitonin (PCT) showed no significant difference (*P* = 0.625) (Fig. 5A).

We next evaluated whether these CSF biomarkers could stratify MTB-positive, non-MTB, and pathogen-negative suspected patients. MTB-positive patients exhibited significantly lower CSF glucose, higher protein, and elevated leukocyte counts compared with both non-MTB and pathogen-negative groups (all *P* < 0.05; Fig. S2). In contrast, patients with pathogen-negative suspected TBM had CSF parameter profiles that were not statistically distinct from those of patients with non-TBM meningitis (all *P* > 0.05), indicating broadly similar CSF inflammatory characteristics.

To distinguish infectious meningitis from noninfectious mimics, we first performed univariate analyses of individual CSF biomarkers. On the training set, CSF protein alone achieved an AUC of 0.797 (95% CI, 0.720–0.874), with a sensitivity of 97.30% (95% CI, 86.18–99.86%) and a specificity of 55.95% (95% CI, 45.31–66.08%). For multivariable modeling, we used a stratified 70/30 split of the cohort into training and test sets. The optimal three-marker model incorporated CSF protein, leukocyte count, and serum CRP. Internal fivefold cross-validation on the training set yielded a mean AUC of 0.817 (95% CI, 0.692–0.942; Fig. S3A). In the test set, the combined model achieved an AUC of 0.877 (95% CI from 1,000 bootstrap resamples: 0.760–0.994), outperforming individual biomarkers, including protein alone (DeLong test, all *P* < 0.05; Fig. 5B and C). For clinical decision-making, we evaluated threshold probabilities in the test set and identified a Youden’s index-optimized cut-off at a predicted probability ≥0.258, which achieved 100.00% sensitivity (95% CI, 72.25–100.00%) and 68.18% specificity (95% CI, 47.32–83.64%). In this cohort, probabilities < 0.258 were associated with high sensitivity for excluding infectious meningitis; however, this threshold requires external validation before clinical rule-out use. In contrast, a high-specificity threshold of ≥0.546 yielded 40.00% sensitivity and 95.45% specificity (95% CI, 77.16–99.88%), providing a conservative criterion for ruling in infectious meningitis. The final model equation is: $P\left(Infection\right)=\frac{1}{1+e^{-\left(0.000800\times Protein+0.023843\times Leukocyte-0.016972\times CRP\right)}}$, where protein is in mg/L; leukocyte is in 10⁶/L; and CRP is in mg/L. No standardization was applied.

Similarly, to distinguish MTB-positive from MTB-negative cases, we first conducted univariate analyses. On the training set, CSF protein alone achieved an AUC of 0.690 (95% CI, 0.593–0.787), with 58.06% sensitivity (95% CI, 47.91–67.58%) and 82.50% specificity (95% CI, 68.05–91.25%). A LASSO model was then developed on the same stratified training set (*n* = 152; 34 MTB-positive, 118 MTB-negative). After excluding cases with missing CRP data, 137 patients (29 MTB-positive, 108 MTB-negative) were included in the LASSO development. Internal fivefold cross-validation on the training set demonstrated robust performance, with a mean AUC of 0.755 (95% CI, 0.631–0.880; Fig. S3B). The three-marker model (leukocyte count, chloride, and CRP) yielded a test AUC of 0.776 (95% bootstrap CI: 0.617–0.934). This combined model outperformed individual biomarkers, including protein alone (DeLong test, all *P* < 0.05; Fig. 5D and E). We also defined three clinically relevant thresholds on the test set: a Youden-optimized cut-off (predicted probability ≥0.754; sensitivity 73.08%, 95% CI, 53.92–86.30%; specificity 75.00%, 95% CI, 46.77–91.11%), a high-sensitivity cut-off (probability ≥0.351; sensitivity 96.15%, specificity 33.33%), and a high-specificity cut-off (probability ≥0.779; sensitivity 50.00%, specificity 91.67%). The final equation is: $P\left(MTB-positive\right)=\frac{1}{1+e^{-\left(0.010205\;\times \;Leukocyte\;-\;0.010031\;\times \;Chloride\;-\;0.010677\;\times \;CRP\right)}}$. All predictors were entered in original clinical units (leukocyte in 10⁶/L, chloride in mmol/L, CRP in mg/L); no standardization was applied.

## DISCUSSION

Meningitis remains a major cause of preventable mortality worldwide, in part because etiologic confirmation is frequently delayed or unobtainable with routine diagnostics. This challenge is amplified for TBM, where clinicians must balance the consequences of delayed treatment against the harms of unnecessary prolonged anti-tuberculous therapy (3, 21). In this study, we validated a highly multiplexed PCR assay (MeltArray CNS) applied to total CSF nucleic acids and evaluated in a prospective cohort of consecutive patients with suspected meningitis. In TB-endemic settings, multiplex CSF testing complements MTB detection by enabling early etiologic clarification across competing meningitis causes that require different therapies. Three findings are notable. First, MeltArray enabled timely pathogen detection across a broad etiologic spectrum, and MTB detection increased with TBM diagnostic certainty, underscoring the limitations of clinical criteria alone (22). Second, among MTB-positive TBM cases, co-detections with other pathogens were common, highlighting etiologic heterogeneity that may influence antimicrobial management (23). Third, routine CSF biomarkers—especially protein—improved discrimination when integrated into multivariable models, supporting their role as adjunctive tools for triage and risk stratification rather than substitutes for pathogen confirmation (24).

Conventional diagnostic methods for TBM have well-documented limitations. AFB smear microscopy provides rapid results but is limited by low sensitivity due to the typically low bacillary burden in CSF, with reported sensitivities around 10% (25). Mycobacterial culture, although considered a reference method, requires prolonged incubation and demonstrates variable sensitivity, with reported yields ranging from approximately 4% to 14% depending on specimen volume, timing of sampling, and prior anti-tuberculous therapy (26). The observed culture positivity in our cohort (8.57%) falls within the previously reported range and reflects these inherent biological and clinical constraints. These limitations underscore the potential value of rapid nucleic acid-based approaches for providing earlier microbiological support in suspected TBM.

In the absence of a single, universally accepted gold standard for TBM diagnosis, current diagnostic classification relies on composite clinical, microbiological, and radiological criteria. Within this framework, the strong gradient in MTB detection across TBM diagnostic categories underscores the limitations of consensus clinical scoring in differentiating TBM from its mimics (20, 22). In our cohort, MeltArray detected MTB in all definite TBM cases, whereas only a minority of probable and possible TBM cases were MTB-positive. This pattern is consistent with the well-recognized heterogeneity of intermediate clinical categories, which may include true TBM with low bacillary burden, alternative infectious etiologies, and noninfectious mimics (22). In high-burden settings, reliance on clinical probability alone may lead to empiric anti-tuberculous therapy in patients without microbiologic evidence while simultaneously delaying appropriate treatment for alternative etiologies (3). Our data support the value of molecular confirmation to strengthen etiologic certainty at the point of initial management and reduce diagnostic delay for MTB-positive disease (27). However, the modest MTB positivity observed in probable and possible TBM highlights the heterogeneity of these categories; the identification of an alternative, syndrome-concordant pathogen may reduce the likelihood of TBM and support an alternative diagnosis, although TBM cannot be excluded in all cases (3, 28). This is where multiplex testing may provide particular clinical value by enabling the identification of alternative treatable etiologies from the same limited CSF specimen.

A key observation was the high frequency of co-detections among MTB-positive TBM cases. Overall, 42.86% of MTB-positive TBM cases had at least one additional pathogen detected, spanning viruses, bacteria, and fungi, and, in some cases, involving multiple organism classes. Such patterns may reflect true polymicrobial CNS infection in selected patients, compartmentalized infection at different sites with spillover into CSF testing, or viral reactivation in the setting of systemic illness and immune perturbation (23, 29–31). We further assessed host immune status in this cohort, including HIV infection and CD4^+^ T-cell counts. HIV infection was not detected among tested patients, while reduced CD4^+^ T-cell counts were observed in both MTB co-detection and monoinfection groups, consistent with previous reports in TBM patients (23). Comparative analysis showed a higher proportion of reduced CD4^+^ T-cell counts in the co-detection group, although this difference was not statistically significant.

Bacterial co-detections in TBM have traditionally been considered rare. Nevertheless, recent molecular studies have reported similar findings in TBM and other central nervous system infections (22, 23). In our cohort, bacterial co-detections were observed, including one case with concurrent detection of MTB and *P. aeruginosa*, consistent with a previous report of MTB/*P. aeruginosa* co-detection in a TBM cohort (23). Importantly, bacterial nucleic acid detection in CSF should be interpreted cautiously, as it may reflect true co-infection, transient or low-level background nucleic acid, or contamination introduced during sampling or processing. Accordingly, MTB-associated bacterial co-detections should be interpreted in conjunction with clinical manifestations, radiological findings, and complementary microbiological evidence.

Viral co-detections showed a similar pattern in MTB-positive TBM cases and should be interpreted cautiously, particularly for neurotropic viruses with alternative disease associations. In our cohort, EBV and JCV detections were evaluated in the context of clinical presentation and radiological findings, and no evidence of EBV-associated lymphoma or JCV-associated progressive multifocal leukoencephalopathy was observed. Nonetheless, these data raise an important clinical consideration: assuming TBM to be uniformly mono-etiologic may overlook additional treatable pathogens in a subset of patients (32). Where co-detections are supported by clinical context (e.g., compatible syndromes, imaging, or corroborative testing), they may justify broader diagnostic evaluation and, in selected cases, expanded antimicrobial coverage (22). Conversely, indiscriminate escalation of therapy based on viral DNA detection alone may increase toxicity without improving outcomes, underscoring the need for quantitative and host-context adjudication in future work (33).

We also found that routine CSF biomarkers provided meaningful adjunctive information, particularly for separating infectious meningitis from noninfectious mimics and stratifying MTB-positive disease versus MTB-negative disease. CSF protein outperformed serum inflammatory markers, consistent with compartmentalized inflammation and blood–brain barrier disruption in CNS infection (24). The multivariable model distinguishing infectious meningitis from noninfectious mimics achieved good discrimination, and the MTB-positive model versus MTB-negative model demonstrated moderate performance. Importantly, these models should be viewed as supportive rather than definitive: several clinically suspected TBM cases were pathogen-negative by MeltArray and had CSF profiles overlapping those of non-TBM meningitis, reinforcing that host–response measures cannot reliably substitute for etiologic confirmation (3). In practice, biomarker-informed probability estimates may help prioritize patients for molecular testing, repeat lumbar puncture, or alternative diagnostic pathways, but should not be used in isolation to initiate or discontinue disease-specific therapy without adequate clinical corroboration and external validation (34).

Our findings also highlight the interpretive challenge of pathogen-negative meningitis. In the prospective cohort, a substantial fraction of meningitis cases remained negative by MeltArray, which likely reflects a mixture of noninfectious etiologies, pathogens not covered by the panel, and false negatives due to low organism burden or sampling limitations (22, 35). In such scenarios, escalation of the diagnostic workup—repeat CSF testing, targeted molecular assays beyond the panel, neuroimaging, or tissue diagnosis when indicated—remains essential (36). These considerations reinforce a diagnostic hierarchy in which pathogen confirmation has primacy, while clinical scoring and biomarker profiles contribute complementary evidence for triage and decision-making (3).

This study has limitations. First, we used a cell-free nucleic acid extraction workflow but did not quantify the relative contribution of cell-associated versus cell-free fractions; therefore, the mechanistic basis of detection (cell-free vs cell-associated) could not be directly distinguished. Second, this study was conducted at a single center, which may limit generalizability to settings with different pathogen epidemiology and pre-test probabilities. Multicenter validation is needed to establish performance across diverse clinical contexts. Third, co-detections were not quantified, and we did not systematically integrate host immune profiling; therefore, we cannot reliably distinguish true co-meningitis from viral reactivation or incidental detection, particularly for EBV (33). In addition, MTB detection by the MeltArray CNS assay is qualitative (positive/negative); therefore, correlation with smear or culture burden was not assessed. Fourth, key host factors that plausibly influence co-detection patterns (e.g., HIV status, diabetes, and immunosuppressive therapy) were not systematically captured, and mechanistic interpretations should, therefore, be considered hypothesis-generating (37). Finally, we did not evaluate longitudinal sampling; serial measurements could clarify dynamic changes in pathogen burden and biomarkers and may improve diagnostic and prognostic modeling (38).

In summary, MeltArray provided timely, broad pathogen detection and high sensitivity for MTB in definite TBM, supporting its role as a rule-in tool for early etiologic confirmation when positive. Frequent co-detections among MTB-positive TBM cases underscore etiologic diversity that may influence antimicrobial management but requires cautious interpretation and prospective validation with quantitative and host-context data. Routine CSF biomarkers add adjunctive value for triage and risk stratification, yet pathogen confirmation remains essential. Together, these findings support an integrated diagnostic approach combining molecular testing with biomarker-informed clinical assessment in settings with a high TBM burden.

## Data Availability

The data underlying this article will be shared on reasonable request to the corresponding author.

## SUPPLEMENTAL MATERIAL

Tables S1 to S4 and Fig. S1 to S3 jcm.00446-26-s0001.pdf Table S1: The pathogen targets of the MeltArray CNS assay. Table S2: Analytical validation of bacterial, fungal, viral, and *M. pneumoniae* targets using the MeltArray CNS assay. Table S3: Comparative diagnostic results of conventional microbiology, targeted PCR, MeltArray CNS assay, and mNGS in 79 clinical CSF samples. Table S4: Individual patient-level diagnostic data corresponding to Table 2 for definite and probable tuberculous meningitis cases. Fig. S1: Analytical performance of the MeltArray CNS assay in detecting target pathogens. Fig. S2: Differential analysis of CSF parameters in patients suspected of having TBM. Fig. S3: Development and cross-validation of biomarker models in the training set for discriminating meningitis etiologies.

ASM does not own the copyrights to Supplemental Material that may be linked to, or accessed through, an article. The authors have granted ASM a non-exclusive, world-wide license to publish the Supplemental Material files. Please contact the corresponding author directly for reuse.
