# Supplementary material for: Etiologic diagnosis of suspected tuberculous meningitis by multiplex PCR of cerebrospinal fluid
Source: J Clin Microbiol. 2026 Aug 20;64(9):e00446-26. doi: 10.1128/jcm.00446-26 (PMC13560704; doi:10.1128/jcm.00446-26)
Supplement: Supplementary file 1 [file jcm.00446-26-s0001.pdf]

**Supplemental Materials for:**

# Etiologic Diagnosis of Suspected Tuberculous Meningitis by Multiplex PCR of Cerebrospinal Fluid

**Supplemental materials include:**

**Table S1.** The pathogen targets of the MeltArray CNS assay.

**Table S2.** Analytical validation of bacterial, fungal, viral, and *M. pneumoniae* targets using the MeltArray CNS assay.

**Table S3.** Comparative diagnostic results of conventional microbiology, targeted PCR, MeltArray CNS assay, and mNGS in 79 clinical CSF samples.

**Table S4.** Individual patient-level diagnostic data corresponding to Table 2 for definite and probable tuberculous meningitis cases.

**Fig. S1.** Analytical performance of the MeltArray CNS assay in detecting target pathogens.

**Fig. S2.** Differential analysis of CSF parameters in patients suspected of having TBM.

**Fig. S3.** Development and cross-validation of biomarker models in the training set for discriminating meningitis etiologies.

**Table S1. The pathogen targets of the MeltArray CNS assay.**

| No. | Target (Full Name) | Abbreviation |
|---|---|---|
| A1 | *Staphylococcus haemolyticus* | *S. haemolyticus* |
| A2 | *Coagulase-Negative Staphylococci* | CoNS |
| A3 | *Pseudomonas aeruginosa* | *P. aeruginosa* |
| A4 | *Staphylococcus hominis* | *S. hominis* |
| A5 | *Enterococcus faecium* | *E. faecium* |
| A6 | *Haemophilus influenzae* | *H. influenzae* |
| A7 | *Pantoea agglomerans* | *P. agglomerans* |
| A8 | *Staphylococcus epidermidis* | *S. epidermidis* |
| A9 | *Enterobacter cloacae* | *E. cloacae* |
| A10 | *Streptococcus pneumoniae* | *S. pneumoniae* |
| A11 | *Listeria monocytogenes* | *L. monocytogenes* |
| A12 | *Klebsiella pneumoniae* | *K. pneumoniae* |
| A13 | *Acinetobacter baumannii* | *A. baumannii* |
| A14 | *Enterobacter aerogenes* | *E. aerogenes* |
| A15 | *Streptococcus suis* | *S. suis* |
| A16 | *Streptococcus agalactiae* | *S. agalactiae* |
| A17 | *Staphylococcus aureus* | *S. aureus* |
| A18 | *Enterococcus faecalis* | *E. faecalis* |
| A19 | *Escherichia coli* K1 | *E. coli* K1 |
| A20 | *Neisseria meningitidis* | *N. meningitidis* |
| B1 | *Serratia marcescens* | *S. marcescens* |
| B2 | *Stenotrophomonas maltophilia* | *S. maltophilia* |
| B3 | *Elizabethkingia meningoseptica* | *E. meningoseptica* |
| B4 | *Pseudomonas putida* | *P. putida* |
| B5 | *Corynebacterium striatum* | *C. striatum* |
| B6 | *Staphylococcus caprae* | *S. caprae* |
| B7 | *Staphylococcus cohnii* | *S. cohnii* |
| B8 | *Klebsiella oxytoca* | *K. oxytoca* |
| B9 | *Streptococcus pyogenes* | *S. pyogenes* |
| B10 | *Staphylococcus warneri* | *S. warneri* |
| B11 | *Cryptococcus* spp. | *Cryptococcus* spp. |
| B12 | *Citrobacter freundii* | *C. freundii* |
| B13 | *Proteus mirabilis* | *P. mirabilis* |
| B14 | *Streptococcus bovis* | *S. bovis* |
| B15 | *Staphylococcus saprophyticus* | *S. saprophyticus* |
| B16 | *Staphylococcus capitis* | *S. capitis* |
| B17 | *Salmonella* spp. | *Salmonella* spp. |
| B18 | *Mycobacterium tuberculosis* | MTB |
| C1 | *Candida albicans* | *C. albicans* |
| C2 | *Candida tropicalis* | *C. tropicalis* |
| C3 | *Pneumocystis jirovecii* | *P. jirovecii* |
| C4 | *Aspergillus niger* | *A. niger* |
| C5 | *Candida parapsilosis* | *C. parapsilosis* |
| C6 | *Candida krusei* | *C. krusei* |
| C7 | *Cryptococcus* spp. | *Cryptococcus* spp. |

| | | |
|---|---|---|
| C8 | *Aspergillus fumigatus* | *A. fumigatus* |
| C9 | *Candida glabrata* | *C. glabrata* |
| C10 | *Rhizopus arrhizus* | *R. arrhizus* |
| C11 | *Aspergillus flavus* | *A. flavus* |
| C12 | *Aspergillus nidulans* | *A. nidulans* |
| C13 | *Aspergillus terreus* | *A. terreus* |
| C14 | *Mucor* spp. | *Mucor* spp. |
| D1 | Epstein-Barr virus | EBV |
| D2 | Human herpesvirus 6 | HHV-6 |
| D3 | Human cytomegalovirus | HCMV |
| D4 | Herpes simplex virus 1 | HSV-1 |
| D5 | Tick-borne encephalitis virus | TBEV |
| D6 | Human parechovirus | HPeV |
| D7 | Adenovirus | ADV |
| D8 | Herpes simplex virus 2 | HSV-2 |
| D9 | Mumps virus | MuV |
| D10 | Human herpesvirus 7 | HHV-7 |
| D11 | Lymphocytic choriomeningitis virus | LCMV |
| D12 | Measles virus | MeV |
| D13 | JC polyomavirus | JCV |
| D14 | Enterovirus | EV |
| D15 | Varicella-zoster virus | VZV |
| D16 | Japanese encephalitis virus | JEV |
| D17 | Rotavirus | RV |
| D18 | Influenza B virus | INFB |
| D19 | Human parvovirus B19 | HPV-B19 |
| D20 | Influenza A virus | INFA |
| E1 | Human bocavirus | HBoV |
| E2 | Human parainfluenza virus 3 | HPIV-3 |
| E3 | *Mycoplasma pneumoniae* | *M. pneumoniae* |
| E4 | Human parainfluenza virus 1 | HPIV-1 |
| E5 | Epstein-Barr virus | EBV |
| E6 | Human metapneumovirus | HMPV |
| E7 | Adenovirus | ADV |
| E8 | Respiratory syncytial virus B | RSV-B |
| E9 | Human rhinovirus | HRV |
| E10 | Influenza B virus | INFB |
| E11 | Respiratory syncytial virus A | RSV-A |
| E12 | Human parainfluenza virus 4 | HPIV-4 |
| E13 | Human parainfluenza virus 2 | HPIV-2 |
| E14 | Human coronavirus NL63 | NL63 |
| E15 | Human coronavirus 229E | 229E |
| E16 | Human coronavirus OC43 | OC43 |
| E17 | Human coronavirus HKU1 | HKU1 |
| E18 | Influenza A virus | INFA |

**Table S2. Analytical validation of bacterial, fungal, viral, and *M. pneumoniae* targets using the MeltArray CNS assay.**

| No. | Microorganism | n | gDNA (copies/μL) | Detection |
|---|---|---|---|---|
| 1 | *E. Coli* K1 | 162 | 1.00×10^3 | 162/162 (100.00%) |
| 2 | *A. baumannii* | 77 | 1.00×10^3 | 77/77 (100.00%) |
| 3 | *H. influenzae* | 40 | 1.00×10^3 | 40/40 (100.00%) |
| 4 | *K. pneumoniae* | 37 | 1.00×10^3 | 37/37 (100.00%) |
| 5 | CoNS | 32 | 1.00×10^3 | 32/32 (100.00%) |
| 6 | *S. epidermidis* | 25 | 1.00×10^3 | 25/25 (100.00%) |
| 7 | *E. faecalis* | 24 | 1.00×10^3 | 24/24 (100.00%) |
| 8 | *E. faecium* | 24 | 1.00×10^3 | 24/24 (100.00%) |
| 9 | *S. aureus* | 21 | 1.00×10^3 | 21/21 (100.00%) |
| 10 | *S. pneumoniae* | 16 | 1.00×10^3 | 16/16 (100.00%) |
| 11 | *P. aeruginosa* | 11 | 1.00×10^3 | 11/11 (100.00%) |
| 12 | *S. haemolyticus* | 11 | 1.00×10^3 | 11/11 (100.00%) |
| 13 | *S. agalactiae* | 8 | 1.00×10^3 | 8/8 (100.00%) |
| 14 | *E. aerogenes* | 7 | 1.00×10^3 | 7/7 (100.00%) |
| 15 | *E. cloacae* | 3 | 1.00×10^3 | 3/3 (100.00%) |
| 16 | *S. hominis* | 3 | 1.00×10^3 | 3/3 (100.00%) |
| 17 | *L. monocytogenes* | 2 | 1.00×10^3 | 2/2 (100.00%) |
| 18 | *P. mirabilis* | 31 | 1.00×10^3 | 31/31 (100.00%) |
| 19 | *Salmonella* spp. | 19 | 1.00×10^3 | 19/19 (100.00%) |
| 20 | *C. freundii* | 12 | 1.00×10^3 | 12/12 (100.00%) |
| 21 | *S. marcescens* | 8 | 1.00×10^3 | 8/8 (100.00%) |
| 22 | *K. oxytoca* | 8 | 1.00×10^3 | 8/8 (100.00%) |
| 23 | *S. saprophyticus* | 5 | 1.00×10^3 | 5/5 (100.00%) |
| 24 | *C. striatum* | 4 | 1.00×10^3 | 4/4 (100.00%) |
| 25 | *S. capitis* | 4 | 1.00×10^3 | 4/4 (100.00%) |

| | | | | |
|---|---|---|---|---|
| 26 | *E. meningoseptica* | 4 | 1.00×10^3 | 4/4 (100.00%) |
| 27 | *P. putida* | 4 | 1.00×10^3 | 4/4 (100.00%) |
| 28 | *S. pyogenes* | 3 | 1.00×10^3 | 3/3 (100.00%) |
| 29 | *S. warneri* | 3 | 1.00×10^3 | 3/3 (100.00%) |
| 30 | *S. bovis* | 2 | 1.00×10^3 | 2/2 (100.00%) |
| 31 | *S. maltophilia* | 2 | 1.00×10^3 | 2/2 (100.00%) |
| 32 | *S. caprae* | 2 | 1.00×10^3 | 2/2 (100.00%) |
| 33 | *S. cohnii* | 1 | 1.00×10^3 | 1/1 (100.00%) |
| 34 | *M. avium** | 2 | 1.00×10^5 | 0/2 (100.00%) |
| 35 | *M. intracellulare** | 2 | 1.00×10^5 | 0/2 (100.00%) |
| 36 | *M. abscessus** | 2 | 1.00×10^5 | 0/2 (100.00%) |
| 37 | *M. fortuitum** | 2 | 1.00×10^5 | 0/2 (100.00%) |
| 38 | *M. kansasii** | 2 | 1.00×10^5 | 0/2 (100.00%) |
| 39 | *M. chelonae** | 2 | 1.00×10^5 | 0/2 (100.00%) |
| 40 | *M. gordonae** | 2 | 1.00×10^5 | 0/2 (100.00%) |
| 41 | *M. xenopi** | 2 | 1.00×10^5 | 0/2 (100.00%) |
| 42 | *M. scrofulaceum** | 2 | 1.00×10^5 | 0/2 (100.00%) |
| 43 | *M. marinum** | 2 | 1.00×10^5 | 0/2 (100.00%) |
| 44 | *C. albicans* | 1 | 1.00×10^3 | 1/1 (100.00%) |
| 45 | *C. tropicalis* | 1 | 1.00×10^3 | 1/1 (100.00%) |
| 46 | *A. niger* | 1 | 1.00×10^3 | 1/1 (100.00%) |
| 47 | *Cryptococcus* spp. | 1 | 1.00×10^3 | 1/1 (100.00%) |
| 48 | *A. fumigatus* | 1 | 1.00×10^3 | 1/1 (100.00%) |
| 49 | *C. glabrata* | 1 | 1.00×10^3 | 1/1 (100.00%) |
| 50 | *R. arrhizus* | 1 | 1.00×10^3 | 1/1 (100.00%) |
| 51 | *C. parapsilosis* | 1 | 1.00×10^3 | 1/1 (100.00%) |
| 52 | *C. krusei* | 1 | 1.00×10^3 | 1/1 (100.00%) |
| 53 | *A. flavus* | 1 | 1.00×10^3 | 1/1 (100.00%) |
| 54 | *A. nidulans* | 1 | 1.00×10^3 | 1/1 (100.00%) |
| 55 | *A. terreus* | 1 | 1.00×10^3 | 1/1 (100.00%) |

| | | | | |
|---|---|---|---|---|
| 56 | *Mucor* spp. | 1 | 1.00×10^3 | 1/1 (100.00%) |
| 57 | *Penicillium** | 1 | 1.00×10^5 | 0/1 (0.00%) |
| 58 | *Alternaria** | 1 | 1.00×10^5 | 0/1 (0.00%) |
| 59 | *Saprolegnia** | 1 | 1.00×10^5 | 0/1 (0.00%) |
| 60 | *Fusarium** | 1 | 1.00×10^5 | 0/1 (0.00%) |
| 61 | *T. Asperellum** | 1 | 1.00×10^5 | 0/1 (0.00%) |
| 62 | HCMV | 3 | 1.00×10^3 | 3/3 (100.00%) |
| 63 | INFA | 3 | 1.00×10^3 | 3/3 (100.00%) |
| 64 | EBV | 2 | 1.00×10^3 | 2/2 (100.00%) |
| 65 | HHV-6 | 2 | 1.00×10^3 | 2/2 (100.00%) |
| 66 | INFB | 3 | 1.00×10^3 | 3/3 (100.00%) |
| 67 | ADV | 2 | 1.00×10^3 | 2/2 (100.00%) |
| 68 | HPIV-3 | 3 | 1.00×10^3 | 3/3 (100.00%) |
| 69 | HPIV-1 | 2 | 1.00×10^3 | 2/2 (100.00%) |
| 70 | HRV | 10 | 1.00×10^3 | 10/10 (100.00%) |
| 71 | RSV-A | 3 | 1.00×10^3 | 3/3 (100.00%) |
| 72 | RSV-B | 3 | 1.00×10^3 | 3/3 (100.00%) |
| 73 | HMPV | 3 | 1.00×10^3 | 3/3 (100.00%) |
| 74 | HBoV | 1 | 1.00×10^3 | 1/1 (100.00%) |
| 75 | OC43 | 3 | 1.00×10^3 | 3/3 (100.00%) |
| 76 | NL63 | 2 | 1.00×10^3 | 2/2 (100.00%) |
| 77 | 229E | 2 | 1.00×10^3 | 2/2 (100.00%) |
| 78 | HPIV-4 | 8 | 1.00×10^3 | 8/8 (100.00%) |
| 79 | HPIV-2 | 8 | 1.00×10^3 | 8/8 (100.00%) |
| 80 | *M. pneumoniae* | 3 | 1.00×10^3 | 3/3 (100.00%) |

A total of 80 microorganisms were evaluated, including 33 bacterial species derived from archived clinical isolates (No. 1–33), 10 non-tuberculous mycobacteria (NTM) species for analytical specificity assessment (No. 34–43), 18 fungal organisms (No. 44–61), and 18 viral targets plus *M. pneumoniae* (No. 62–80). Viral and *M. pneumoniae* targets were tested using pre-characterized clinical bronchoalveolar lavage fluid (BALF) specimens. Among fungal organisms, 13 were panel-included targets, whereas 5 were non-target species used for exclusivity testing. NTM species and non-target fungal organisms are indicated by an asterisk (*). All bacterial targets, panel-included fungal targets, and viral/*M. pneumoniae* targets were tested at $10^3$ copies/μL, while NTM species and non-target fungal organisms were tested at $10^5$ copies/μL for analytical specificity assessment. Results are expressed as n/N (%), representing correctly detected samples/total tested. 0/N indicates no cross-reactivity under the tested conditions.

**Table S3. Comparative diagnostic results of conventional microbiology, targeted PCR, MeltArray CNS assay, and mNGS in 79 clinical CSF samples.**

| Patient ID | Group | Prior antimicrobial therapy | AFB smear | Routine culture | Mycobacterial culture | MTB-specific PCR | MeltArray | mNGS |
|---|---|---|---|---|---|---|---|---|
| Patient 1 | Non-TBM | No | - | - | - | - | *K. pneumoniae, C. freundii,* JCV | *K. pneumoniae, C. freundii* |
| Patient 2 | Non-TBM | Yes | - | - | - | - | *C. freundii,* EBV | *C. freundii,* EBV |
| Patient 3 | Possible TBM | Yes | - | - | - | - | *S. warneri,* HHV-6 | *S. warneri,* *HHV-8 |
| Patient 4 | Probable TBM | Yes | - | - | - | - | MTB, JCV | MTB |
| Patient 5 | Non-TBM | Yes | - | - | - | - | *S. caprae* | *S. caprae* |
| Patient 6 | Definite TBM | Yes | - | - | - | + | MTB, *P. aeruginosa* | MTB, *P. aeruginosa* |
| Patient 7 | Non-TBM | No | - | - | - | - | *P. aeruginosa* | *P. aeruginosa* |
| Patient 8 | Probable TBM | Yes | - | - | - | - | *P. aeruginosa, S. warneri,* EBV | *P. aeruginosa, S. warneri,* EBV |
| Patient 9 | Definite TBM | Yes | - | - | - | + | MTB, EBV | MTB, EBV |
| Patient 10 | Possible TBM | Yes | - | - | - | - | *S. caprae,* EBV | *S. caprae* |
| Patient 11 | Possible TBM | Yes | - | - | - | - | *K. aerogene,* EBV | *K. aerogenes* |
| Patient 12 | Definite TBM | Yes | - | - | - | + | MTB, EBV | EBV |
| Patient 13 | Possible TBM | Yes | - | - | - | - | *S. epidermidis, K. aerogenes,* EBV | *S. epidermidis, K. aerogenes* |
| Patient 14 | Possible TBM | Yes | - | - | - | - | *E. meningoseptica* | *E. meningoseptica* |
| Patient 15 | Possible TBM | Yes | - | - | - | - | *A. fumigatus* | *A. fumigatus* |
| Patient 16 | Possible TBM | Yes | - | - | - | - | *E. faecalis, C. glabrata,* JCV, JEV | *E. faecalis* |
| Patient 17 | Possible TBM | Yes | - | - | - | - | *A. fumigatus,* JCV | *A. fumigatus,* JCV |
| Patient 18 | Probable TBM | Yes | - | - | - | - | *E. coli* K1, JCV | *E. coli* K1, *HIV-1 |
| Patient 19 | Definite TBM | Yes | - | - | + | - | MTB, EBV | MTB, EBV |
| Patient 20 | Non-TBM | Yes | - | - | - | - | - | - |

| | | | | | | | | |
|---|---|---|---|---|---|---|---|---|
| Patient 21 | Possible TBM | No | - | - | - | - | - | - |
| Patient 22 | Definite TBM | Yes | - | - | - | - | MTB | MTB |
| Patient 23 | Possible TBM | Yes | - | - | - | - | - | *HIV-1 |
| Patient 24 | Definite TBM | Yes | - | - | - | + | MTB, EBV | MTB, EBV |
| Patient 25 | Definite TBM | Yes | - | - | - | + | MTB, ADV | MTB |
| Patient 26 | Definite TBM | Yes | - | - | - | + | *E. faecalis*, MTB | *E. faecalis*, MTB |
| Patient 27 | Non-TBM | Yes | - | - | - | - | *E. faecalis, K. pneumoniae*, JCV | *E. faecalis, K. pneumoniae* |
| Patient 28 | Possible TBM | Yes | - | - | - | - | *S. aureus*, EBV | *S. aureus*, EBV |
| Patient 29 | Possible TBM | Yes | - | - | - | - | JCV | - |
| Patient 30 | Definite TBM | Yes | - | - | - | + | MTB, HHV-7 | MTB, HHV-7 |
| Patient 31 | Definite TBM | Yes | - | - | - | + | MTB, JCV | MTB |
| Patient 32 | Possible TBM | Yes | - | - | - | - | *S. aureus*, EBV | *S. aureus*, EBV |
| Patient 33 | Possible TBM | Yes | - | - | - | - | *S. aureus* | *S. aureus* |
| Patient 34 | Possible TBM | Yes | - | - | - | - | *E. faecalis* | *E. faecalis* |
| Patient 35 | Definite TBM | Yes | - | - | + | - | MTB | MTB |
| Patient 36 | Definite TBM | No | - | - | - | + | MTB, JCV, EBV | MTB, EBV |
| Patient 37 | Definite TBM | Yes | - | - | - | + | MTB, ADV | MTB |
| Patient 38 | Possible TBM | Yes | - | - | - | - | EBV, MTB, JCV | - |
| Patient 39 | Possible TBM | Yes | - | - | - | - | JCV | - |
| Patient 40 | Possible TBM | Yes | - | - | - | - | *A. fumigatus* | *A. fumigatus* |
| Patient 41 | Definite TBM | Yes | - | - | - | + | MTB, *A. niger* | - |
| Patient 42 | Definite TBM | Yes | - | - | - | + | MTB | - |
| Patient 43 | Definite TBM | Yes | - | - | - | + | MTB | - |
| Patient | Definite | Yes | - | - | - | + | MTB | - |

| | | | | | | | | |
|---|---|---|---|---|---|---|---|---|
| 44 | TBM | | | | | | | |
| Patient 45 | Non-TBM | Yes | - | - | - | - | - | - |
| Patient 46 | Possible TBM | Yes | - | - | - | - | - | - |
| Patient 47 | Possible TBM | Yes | - | - | - | - | - | - |
| Patient 48 | Definite TBM | Yes | + | - | + | + | MTB | MTB |
| Patient 49 | Definite TBM | Yes | - | - | - | + | MTB | MTB |
| Patient 50 | Non-meningitis | Yes | - | - | - | - | - | - |
| Patient 51 | Probable TBM | Yes | - | - | - | - | MTB, EBV | MTB, EBV |
| Patient 52 | Possible TBM | Yes | - | - | - | - | JCV | JCV |
| Patient 53 | Probable TBM | Yes | - | - | - | - | EBV | - |
| Patient 54 | Possible TBM | Yes | - | - | - | - | - | - |
| Patient 55 | Non-meningitis | Yes | - | - | - | - | - | - |
| Patient 56 | Possible TBM | Yes | - | - | - | - | *C. albicans* | *C. albicans* |
| Patient 57 | Non-TBM | No | - | - | - | - | *C. krusei* | - |
| Patient 58 | Non-meningitis | Yes | - | - | - | - | - | - |
| Patient 59 | Possible TBM | Yes | - | - | - | - | - | - |
| Patient 60 | Possible TBM | No | - | - | - | - | - | - |
| Patient 61 | Definite TBM | Yes | - | - | - | + | MTB | - |
| Patient 62 | Possible TBM | Yes | - | - | - | - | - | - |
| Patient 63 | Possible TBM | Yes | - | - | - | - | - | - |
| Patient 64 | Possible TBM | Yes | - | - | - | - | EBV | EBV |
| Patient 65 | Possible TBM | Yes | - | - | - | - | - | - |
| Patient 66 | Probable TBM | Yes | - | - | - | - | EBV | EBV |
| Patient 67 | Non-meningitis | Yes | - | - | - | - | - | - |

| | | | | | | | | |
|---|---|---|---|---|---|---|---|---|
| Patient 68 | Non-meningitis | Yes | - | - | - | - | - | - |
| Patient 69 | Probable TBM | Yes | - | - | - | - | EBV | EBV |
| Patient 70 | Definite TBM | Yes | - | - | - | + | MTB | - |
| Patient 71 | Probable TBM | Yes | - | - | - | - | EBV | EBV |
| Patient 72 | Probable TBM | Yes | - | - | - | - | JCV | - |
| Patient 73 | Non-TBM | No | - | - | - | - | *C. freundii, P. putida,* JCV | JCV |
| Patient 74 | Non-TBM | Yes | - | - | - | - | EBV | - |
| Patient 75 | Probable TBM | Yes | - | - | - | - | EBV | EBV |
| Patient 76 | Possible TBM | Yes | - | - | - | - | JCV | - |
| Patient 77 | Non-TBM | Yes | - | - | - | - | EBV | EBV |
| Patient 78 | Possible TBM | Yes | - | - | - | - | - | - |
| Patient 79 | Probable TBM | Yes | - | - | - | - | MTB, EBV, JCV | EBV, JCV |

Routine culture refers to routine bacterial and fungal cultures performed on blood/chocolate agar and Sabouraud dextrose agar. Mycobacterial culture refers to MTB culture performed using BD BACTEC MGIT 960 and Löwenstein–Jensen medium. mNGS, metagenomic next-generation sequencing; "+" indicates a positive result; "-" indicates a negative result. *HHV-8 and *HIV-1 are not included in the MeltArray CNS detection panel.

**Table S4. Individual patient-level diagnostic data corresponding to Table 2 for definite and probable tuberculous meningitis cases.**

| Patient ID | Group | Prior antimicrobial therapy | AFB smear | Mycobacterial culture | MTB-specific PCR | MeltArray |
|---|---|---|---|---|---|---|
| Patient 9 | Definite | Yes | - | - | + | JCV, MTB, *E. coli* K1, *A. baumannii*, *E. faecium* |
| Patient 16 | Definite | Yes | - | - | + | MTB |
| Patient 17 | Definite | Yes | - | - | + | MTB |
| Patient 18 | Definite | Yes | - | - | + | MTB |
| Patient 19 | Definite | Yes | - | - | + | EBV, MTB |
| Patient 24 | Probable | Yes | - | - | - | *P. aeruginosa, S. warneri* |
| Patient 25 | Definite | Yes | - | - | + | MTB, *P. aeruginosa* |
| Patient 35 | Definite | Yes | - | - | + | MTB |
| Patient 42 | Definite | Yes | - | - | + | EBV, MTB |
| Patient 47 | Definite | Yes | - | - | + | EBV, MTB |
| Patient 70 | Probable | Yes | - | - | - | EBV, *S. caprae*, *E. coli* K1 |
| Patient 72 | Definite | Yes | - | - | + | *E. faecalis,* MTB |
| Patient 73 | Probable | Yes | - | - | - | - |
| Patient 79 | Probable | Yes | - | - | - | - |
| Patient 80 | Probable | Yes | - | - | - | - |
| Patient 84 | Probable | Yes | - | - | - | ADV, *E. coli* K1 |
| Patient 86 | Probable | Yes | - | - | - | JCV, *E. coli* K1 |
| Patient 87 | Probable | Yes | - | - | - | EBV |
| Patient 88 | Probable | Yes | - | - | - | JCV, *S. epidermidis* |
| Patient 91 | Probable | Yes | - | - | - | JCV, EBV |
| Patient 92 | Probable | Yes | - | - | - | JCV, *E. coli* K1, *S. aureus* |
| Patient 93 | Definite | Yes | - | - | + | MTB*, A. niger* |
| Patient 97 | Probable | Yes | - | - | - | JCV, HHV-6, EBV |
| Patient 101 | Probable | Yes | - | - | - | - |
| Patient 102 | Probable | Yes | - | - | - | JCV, MTB |
| Patient 105 | Definite | Yes | - | - | + | *S. aureus,* MTB |
| Patient 110 | Probable | Yes | - | - | - | JCV |
| Patient 111 | Definite | Yes | - | - | + | EBV, MTB |
| Patient 113 | Definite | No | - | - | - | MTB |
| Patient 118 | Probable | Yes | - | - | - | MTB |
| Patient 128 | Probable | Yes | - | - | - | - |
| Patient 130 | Probable | Yes | - | - | - | - |
| Patient 131 | Probable | Yes | - | - | - | - |
| Patient 138 | Probable | Yes | - | - | - | - |
| Patient 139 | Definite | Yes | - | - | + | MTB |
| Patient 141 | Definite | Yes | - | - | + | MTB |
| Patient 143 | Probable | Yes | - | - | - | EBV |
| Patient 145 | Definite | Yes | - | - | + | MTB |
| Patient 146 | Probable | Yes | - | - | - | - |
| Patient 163 | Definite | Yes | + | + | - | EBV, MTB |
| Patient 167 | Definite | No | + | + | - | MTB |

| | | | | | | |
|---|---|---|---|---|---|---|
| Patient 171 | Probable | Yes | - | - | - | *C. parapsilosis* |
| Patient 176 | Probable | Yes | - | - | - | EBV |
| Patient 177 | Probable | Yes | - | - | - | JCV |
| Patient 179 | Definite | Yes | - | - | + | EBV, MTB |
| Patient 180 | Definite | Yes | - | - | + | MTB |
| Patient 181 | Probable | Yes | - | - | - | HHV-6 |
| Patient 183 | Probable | Yes | - | - | - | - |
| Patient 189 | Probable | Yes | - | - | - | EBV |
| Patient 191 | Probable | Yes | - | - | - | *C. albicans* |
| Patient 196 | Probable | Yes | - | - | - | EBV, *C. parapsilosis* |
| Patient 197 | Definite | Yes | - | - | + | MTB |
| Patient 200 | Probable | Yes | - | - | - | - |
| Patient 201 | Probable | Yes | - | - | - | EBV |
| Patient 203 | Probable | Yes | - | - | - | HHV-7, EBV |
| Patient 205 | Definite | Yes | - | - | + | MTB |
| Patient 206 | Probable | Yes | - | - | - | - |
| Patient 207 | Probable | Yes | - | - | - | EBV |
| Patient 209 | Probable | Yes | - | - | - | - |
| Patient 213 | Probable | Yes | - | - | - | - |
| Patient 214 | Definite | Yes | - | - | + | MTB |
| Patient 215 | Probable | No | - | - | - | MTB |
| Patient 220 | Definite | Yes | + | + | + | MTB |
| Patient 221 | Definite | Yes | - | - | + | MTB |
| Patient 222 | Definite | Yes | + | - | + | MTB |
| Patient 223 | Probable | Yes | - | - | - | - |
| Patient 226 | Probable | Yes | - | - | - | *S. caprae* |
| Patient 227 | Definite | Yes | - | - | + | MTB |
| Patient 230 | Definite | Yes | - | - | + | MTB |
| Patient 233 | Probable | Yes | - | - | - | MTB |
| Patient 234 | Probable | Yes | - | - | - | MTB |
| Patient 238 | Probable | No | - | - | - | - |
| Patient 239 | Definite | Yes | - | - | + | MTB |
| Patient 240 | Definite | Yes | - | - | + | MTB, *S. epidermidis* |
| Patient 241 | Definite | Yes | - | - | + | MTB, *C. parapsilosis* |
| Patient 242 | Probable | Yes | - | - | - | *S. aureus* |
| Patient 245 | Definite | No | - | - | + | MTB |
| Patient 246 | Probable | Yes | - | - | - | EBV, MTB |
| Patient 247 | Definite | Yes | - | - | + | MTB, *S. aureus, S. bovis* |
| Patient 248 | Probable | Yes | - | - | - | - |
| Patient 251 | Probable | Yes | - | - | - | *S. warneri* |
| Patient 252 | Probable | Yes | - | - | - | *C. albicans* |
| Patient 253 | Probable | Yes | - | - | - | EBV, MTB |
| Patient 254 | Definite | Yes | - | - | + | MTB |

"+" indicates a positive result; "-" indicates a negative result.

**Fig. S1**

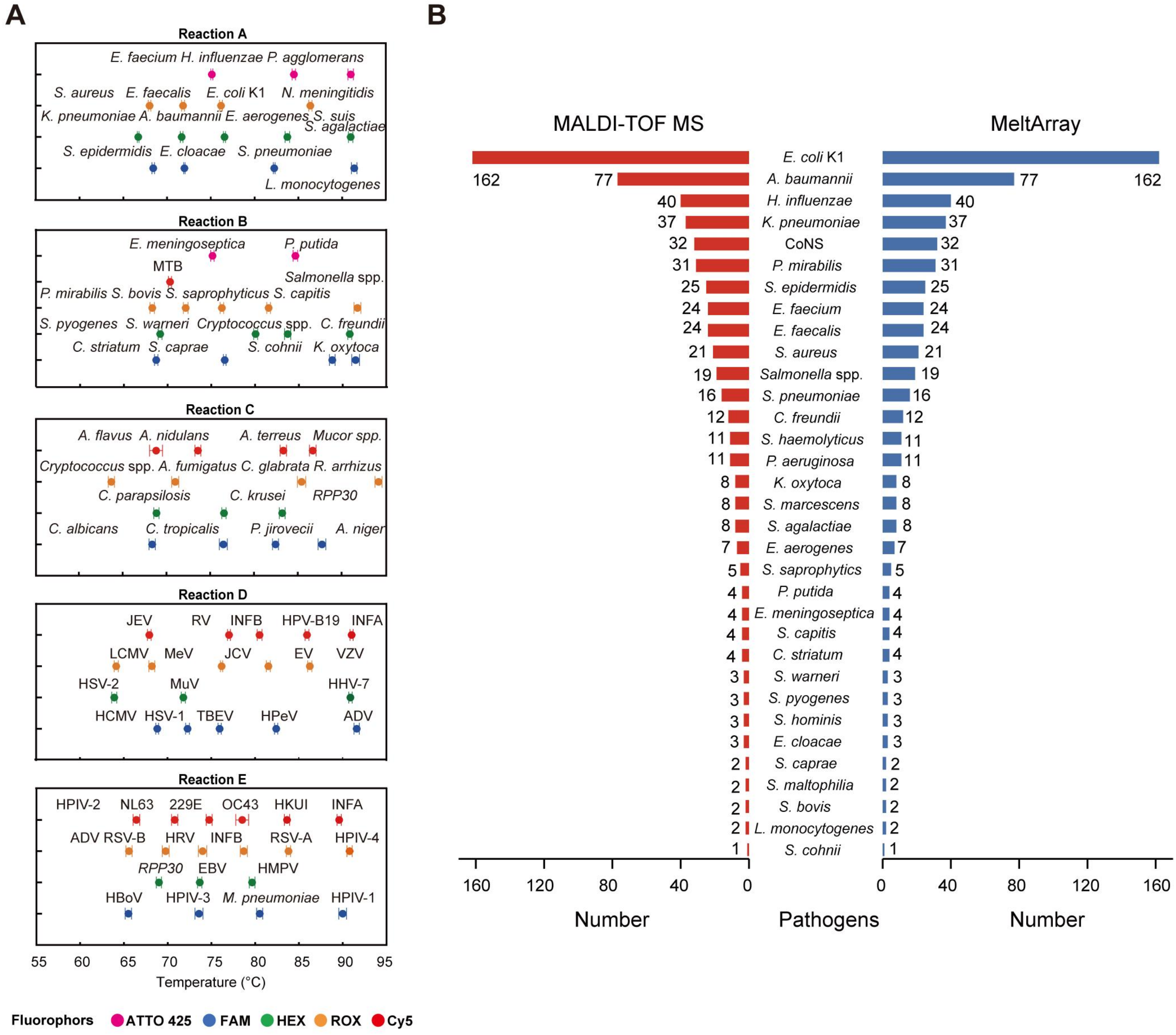


**Fig. S1. Analytical performance of the MeltArray CNS assay in detecting target pathogens.** (A) Reproducibility assessment of the MeltArray CNS assay based on $T_m$ value distributions, using Mean±3SD as the acceptable range for $T_m$ value variation of each target. (B) Accuracy evaluation of the MeltArray CNS assay using 615 clinical pathogen isolates, compared to MALDI-TOF MS assays.

**Fig. S2**

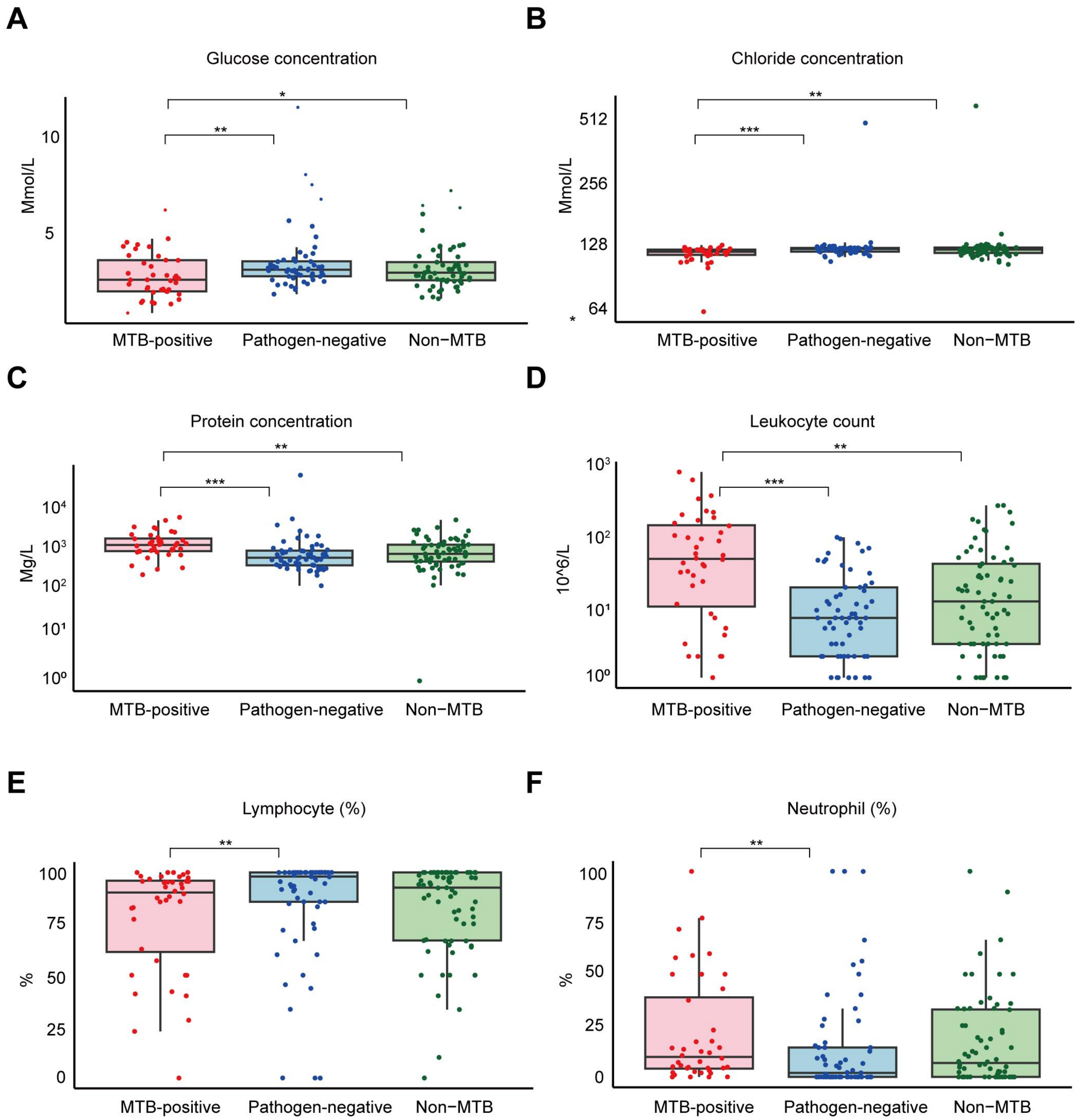


**Fig. S2. Differential analysis of CSF parameters in patients suspected of having TBM.** Analysis of (A) glucose concentration, (B) chloride concentration, (C) protein concentration, (D) leukocyte count, (E) lymphocyte percentage, and (F) neutrophil percentage. "MTB-positive" refers to suspected TBM patients with a MeltArray result positive for MTB, “pathogen-negative" indicates suspected TBM patients with a negative MeltArray result, and "non-MTB" denotes suspected TBM patients with a MeltArray result positive for a pathogen other than MTB. Statistical significance assessed by Mann–Whitney U test (* $p < 0.05$, ** $p < 0.01$, *** $p < 0.001$).

**Fig. S3**

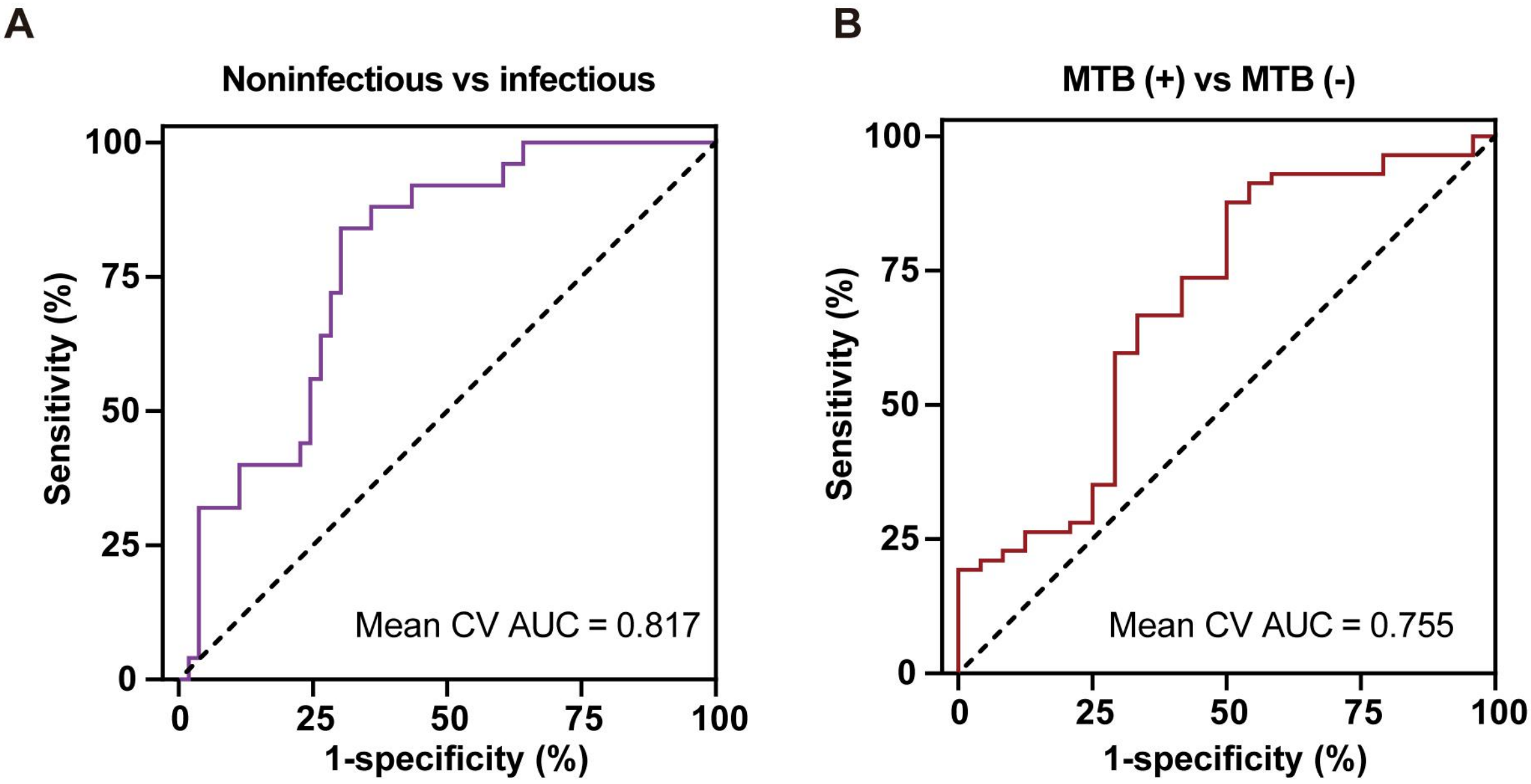


**Fig. S3. Development and cross-validation of biomarker models in the training set for discriminating meningitis etiologies.** Cross-validation of the optimized integrated models was performed within the training set. Mean receiver operating characteristic (ROC) curves, derived from stratified 5-fold cross-validation within the training set, are presented for two diagnostic tasks: (A) discriminating noninfectious mimics from infectious meningitis using a three-marker model (protein, leukocyte count, and CRP); and (B) discriminating MTB-positive from MTB-negative meningitis using a three-marker model (leukocyte count, chloride, and CRP). The dashed line in each panel represents the line of no discrimination (AUC = 0.5).
